# Ultrasensitive profiling of UV-induced mutations identifies thousands of subclinical facial tumors in tuberous sclerosis complex

**DOI:** 10.1172/JCI155858

**Published:** 2022-05-16

**Authors:** Katarzyna Klonowska, Joannes M. Grevelink, Krinio Giannikou, Barbara A. Ogorek, Zachary T. Herbert, Aaron R. Thorner, Thomas N. Darling, Joel Moss, David J. Kwiatkowski

**Affiliations:** 1 Cancer Genetics Laboratory, Division of Pulmonary and Critical Care Medicine, Brigham and Women’s Hospital, Harvard Medical School, Boston, Massachusetts, USA.; 2 Boston Dermatology and Laser Center, Massachusetts General Hospital, Boston, Massachusetts, USA.; 3 Molecular Biology Core Facilities and; 4 Center for Cancer Genomics, Dana-Farber Cancer Institute, Boston, Massachusetts, USA.; 5 Department of Dermatology, Uniformed Services University, Bethesda, Maryland, USA.; 6 Pulmonary Branch, National Heart, Lung, and Blood Institute, National Institutes of Health, Bethesda, Maryland, USA.

**Keywords:** Dermatology, Genetics, Molecular genetics, Skin cancer, Tumor suppressors

## Abstract

**Background:**

Tuberous sclerosis complex (TSC) is a neurogenetic syndrome due to loss-of-function mutations in *TSC2* or *TSC1*, characterized by tumors at multiple body sites, including facial angiofibroma (FAF). Here, an ultrasensitive assessment of the extent and range of UV-induced mutations in TSC facial skin was performed.

**Methods:**

A multiplex high-sensitivity PCR assay (MHPA) was developed, enabling mutation detection at extremely low (<0.1%) variant allele frequencies (VAFs).

**Results:**

MHPA assays were developed for both *TSC2* and *TP53*, and applied to 81 samples, including 66 skin biopsies. UV-induced second-hit mutation causing inactivation of *TSC2* was pervasive in TSC facial skin with an average of 4.8 mutations per 2-mm biopsy at median VAF 0.08%, generating more than 150,000 incipient facial tumors (subclinical “micro-FAFs”) in the average TSC subject. The MHPA analysis also led to the identification of a refined UV-related indel signature and a recurrent complex mutation pattern, consisting of both a single-nucleotide or dinucleotide variant and a 1- to 9-nucleotide deletion, in *cis*.

**Conclusion:**

TSC facial skin can be viewed as harboring a patchwork of clonal fibroblast proliferations (micro-FAFs) with indolent growth, a small proportion of which develop into clinically observable FAF. Our observations also expand the spectrum of UV-related mutation signatures.

**Funding:**

This work was supported by the TSC Alliance; the Engles Family Fund for Research in TSC and LAM; and the NIH, National Heart, Lung, and Blood Institute (U01HL131022-04 and Intramural Research Program).

## Graphical abstract

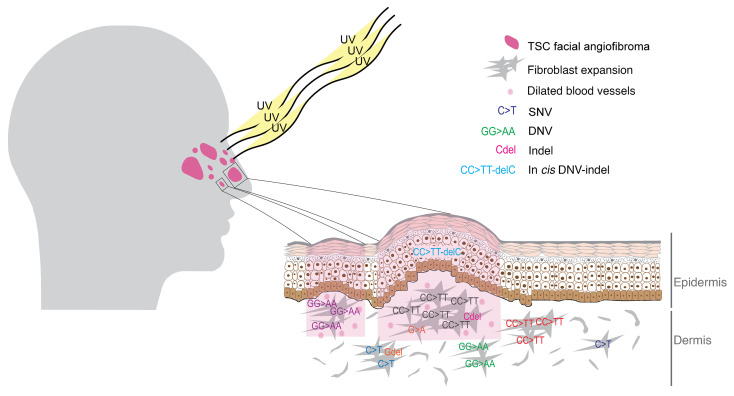

## Introduction

Tuberous sclerosis complex (TSC) is a relatively common (incidence 1 in 6000 live births) autosomal dominant tumor suppressor gene syndrome, with prominent brain manifestations. TSC is due to loss-of-function germline heterozygous or systemic mosaic variants in either of 2 tumor suppressor genes, *TSC2* or *TSC1*. Multiple distinctive hamartomatous tumors develop in multiple tissues and organs in TSC, including skin (facial angiofibroma [FAF]), brain (subependymal giant cell astrocytoma), heart (rhabdomyoma), kidney (angiomyolipoma), and lungs (lymphangioleiomyomatosis) (1). TSC tumors develop following the Knudson 2-hit tumor suppressor gene model (2–9), with a second hit in *TSC1*/*TSC2* occurring through different genetic mechanisms, including copy-neutral loss of heterozygosity (LOH) and second-hit point mutations (indels/single-nucleotide variants [SNVs]).

Although most TSC tumors are composed largely of tumor cells with complete *TSC1*/*TSC2* loss, TSC FAFs occur in the dermis with nontumor vascular, inflammatory, and other components. TSC1/TSC2-null fibroblasts are the core tumor cells in TSC FAFs (10). However, due to these other cell types, and the overlying keratinocyte-rich epidermis, biopsies of FAF have identified second-hit mutations at relatively low variant allele frequencies (VAFs) in *TSC1*/*TSC2*, always less than 10% and often less than 2% (11, 12).

We have performed a series of massively parallel sequencing (MPS) studies on TSC subjects and shown that low-level generalized mosaicism (involving multiple tissues and TSC tumors; median VAF in blood: 1.7% [ref. 11]) affects a substantial subset (10%–15%) of individuals diagnosed with TSC and is especially common in TSC patients who had no mutation identified by conventional genetic testing in clinical laboratories (11–13). FAF biopsies typically contain higher levels of the mosaic pathogenic allele than corresponding blood or normal skin, due to enrichment for clonal tumor fibroblasts with 2-hit inactivation of either *TSC2* or *TSC1* (11), despite the cell admixture noted above. MPS sequencing of short-term cultures of fibroblast-like tumor cells from 29 TSC skin tumors, including FAFs and other TSC skin lesions, showed that 89% of the second-hit mutations were small indels or SNVs. Seven of 14 (50%) *TSC2* somatic mutations were CC:GG>TT:AA transitions, a mutation known to be due to UV radiation–induced DNA damage, and were identified exclusively in FAF fibroblast cell cultures from sun-exposed sites (14). Our subsequent MPS analyses of a total of 55 FAF biopsies revealed 18 second-hit mutations, including 12 of 18 (66%) CC:GG>TT:AA or C:G>T:A mutations, consistent with UV-induced mutation (11, 12). In 1 of the FAF biopsies (11) and 3 FAF fibroblast cell cultures (14), 2 alternative second-hit mutations were found, suggesting the presence of 2 FAF clones. Thirty-eight of 55 (69%) FAF biopsies (11, 12) had no second-hit mutation identified, consistent with any of 3 possibilities: (a) the biopsy was not deep enough to capture dermal fibroblast DNA or the clinical diagnosis of FAF was incorrect; (b) LOH was the mechanism of second hit, not easily identified by our methods of analysis for these FAF biopsies; (c) second-hit mutant allele frequencies were too low for detection (<1% VAF) by our previous method.

Simultaneously, the prevalence of UV-induced mutations and their effect on the keratinocyte and other cell populations have been defined in multiple MPS analyses of epidermis from normal skin and skin cancer biopsies (15–18).

**Figure 1:**
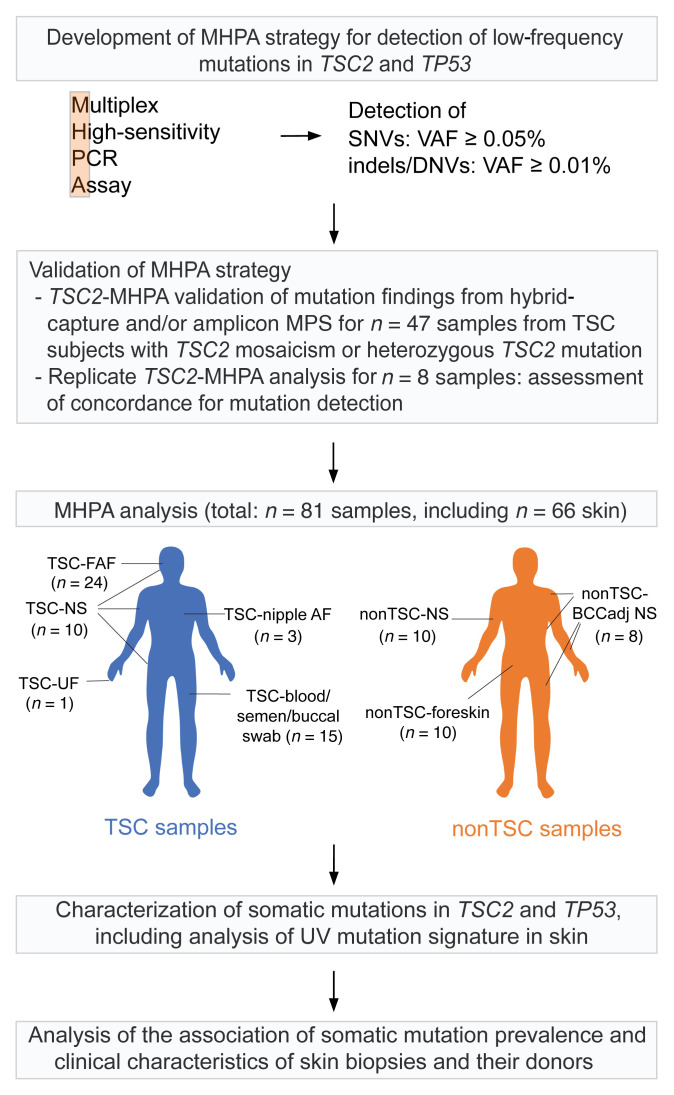
Flow diagram outlining the study design and analyzed samples. The development of the multiplex high-sensitivity PCR assay (MHPA) is described briefly, including validation. The distribution of TSC and non-TSC samples studied according to body region is shown. DNV, dinucleotide variant; indel, insertion/deletion; MPS, massively parallel sequencing; SNV, single-nucleotide variant; TSC, tuberous sclerosis complex; TSC-FAF, TSC facial angiofibroma biopsies; TSC-nipple AF, TSC nipple angiofibroma biopsies; TSC-NS, TSC normal skin; TSC-blood/semen/buccal swab, samples from TSC blood, semen, and buccal swab; TSC-UF, TSC ungual fibroma biopsies; nonTSC-BCCadj NS, normal skin from individuals without TSC, adjacent to resected basal cell carcinoma lesions; nonTSC-NS, normal skin from individuals without TSC, from upper inner arm; nonTSC-foreskin, neonatal foreskin from individuals without TSC; VAF, variant allele frequency.

To examine the extent and range of effects of UV-induced mutation in the facial skin of TSC individuals, we developed an MPS-based method, multiplex high-sensitivity PCR assay (MHPA), with sensitivity of 0.01% to 0.05% VAF for most *TSC2* mutations. MHPA analysis led to the discovery that UV-induced mutations causing inactivation of *TSC2* are pervasive in facial skin of individuals with TSC, generating hundreds of thousands of fibroblast proliferations and incipient facial tumors (subclinical “micro-FAFs”) due to second-hit mutations in *TSC2*, a small proportion of which develop into observable FAF lesions. Our expanded MHPA analysis of both *TSC2* and *TP53*, in a panel of TSC FAFs and normal skin from different body sites, led to the discovery of an extremely high burden of UV-induced mutations in human skin, with greater numbers of variants and at lower VAFs than previously reported (Figure 1 and refs. 15, 18, 19).

## Results

### Development and validation of an MHPA strategy for sequencing error suppression and detection of low-frequency mutations in TSC2 and TP53.

**Figure 2:**
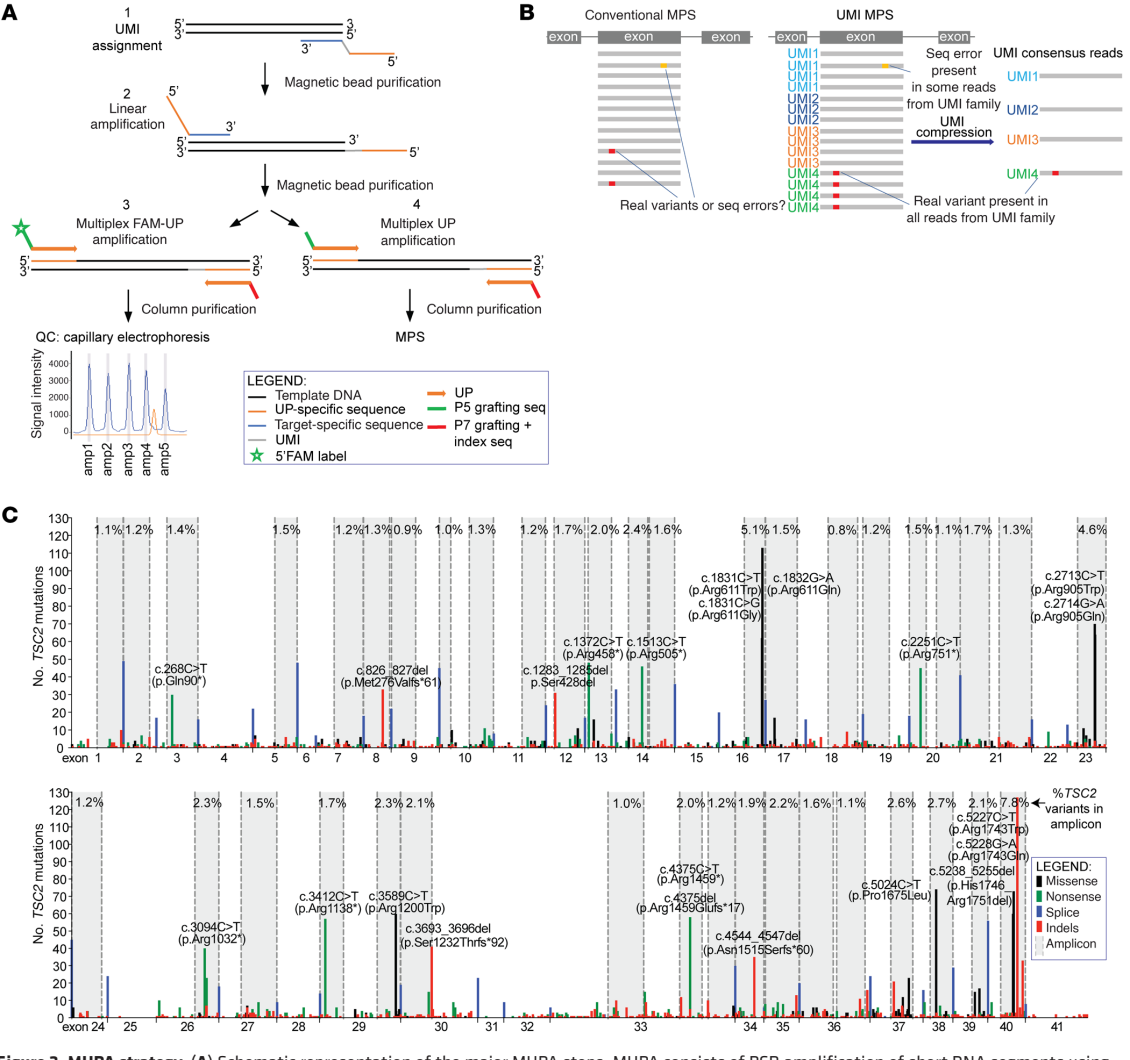
MHPA strategy. (**A**) Schematic representation of the major MHPA steps. MHPA consists of PCR amplification of short DNA segments using primers that include unique molecular identifier (UMI) barcodes, followed by library preparation and sequencing. In the first reaction (step 1), a multiplex linear amplification of each genomic region occurs using reverse primers only with inclusion of a random 14-nt UMI. Following purification, another linear amplification (step 2) of each genomic region occurs using forward primers only. Following purification, amplification of the UMI-barcoded molecules occurs using universal primers (UP) (steps 3 and 4). Step 3 is performed for optimization of MHPA assays, in which amplicons are labeled using a fluorescent dye, 6-carboxyfluorescein (FAM), followed by capillary electrophoresis to assess abundance of each amplicon. Step 4 is used to generate the MHPA libraries, which are purified and subjected to MPS. (**B**) Comparison of conventional and UMI-based MPS variant calling strategies. Barcoding of single DNA molecules with UMIs enables compression of the MHPA data to consensus reads that permits sequencing error suppression. (**C**) Map of deleterious germline *TSC2* variants reported in the LOVD database. The *y* axis indicates the number of *TSC2* variants at a single nucleotide position. Recurrent variants appear as vertical lines. The color of the line indicates the type of variant, as shown in the inset legend. Hotspots with variants reported more than 30 times are labeled with coding sequence nucleotide (c.) and amino acid (p.) position. Splice mutations are summed and shown as a single bar at each exon-exon junction. Genomic regions covered by MHPA amplicons are marked in gray, with indication of the fraction of the deleterious germline variants covered by each of the amplicons (%).

Detection of low–allele frequency (<1%) mutations is challenging because of the high error rate/intrinsic noise in standard MPS (20–22). To obtain reliable detection of extremely-low-VAF (<0.1%) mutations, we developed an ultrasensitive MHPA strategy derived from similar efforts (22–24) (Figure 2A; and see Methods). MHPA is a multiplex amplicon-based strategy that employs barcoding of single DNA molecules with unique molecular identifiers (UMIs), which are random 14-nt sequences, to enable error suppression. MPS read data are compressed to paired-end consensus reads from all the reads for each individual UMI. UMI compressed consensus sequences are retained when there is no variation in the sequencing data among different reads, and converted to the reference sequence when there is inconsistent variation among reads suggestive of PCR error (Figure 2B).

To implement MHPA analysis of *TSC2*, we reviewed the Leiden Open Variation Database (LOVD) (25) and prepared a comprehensive list of pathogenic variants in *TSC2* (total *n =* 4402; unique *n =* 1595) (1). Our goal was to maximize coverage of *TSC2* with relatively uniform read depth, while minimizing the amount of DNA needed for the MHPA reactions. Forty short (48–117 nt) amplicons (queried sequence, Supplemental Table 1.1; supplemental material available online with this article; https://doi.org/10.1172/JCI155858DS1) were designed to amplify *TSC2* exon and exon-intron junction regions with the most mutations, covering in aggregate 74% of reported germline pathogenic *TSC2* variants (Figure 2C and Supplemental Table 1.1). PCR amplification was performed in two 20-plex reactions, to conserve DNA and effectively amplify adjacent genomic regions. After initial first-strand and second-strand linear amplification reactions, universal primer amplification was performed (Figure 2A).

An MHPA assay for *TP53* was also generated, using 12 primer pairs to amplify similar short segments (90–126 nt; queried sequence, Supplemental Table 1.2) of the exons and exon-intron junctions of *TP53*, covering all hotspot regions and 93% of somatic nonsynonymous *TP53* variants, identified previously in normal keratinocytes and skin cancers (Supplemental Table 1.2 and Supplemental Figure 1). Similarly to PCR amplification for the *TSC2*-MHPA assay, PCR amplification for the *TP53*-MHPA assay was performed in 2 separate reactions (each 6-plex).

The *TSC2*-MHPA and *TP53*-MHPA assays were used for the analysis of 81 and 39 DNA samples, respectively (Table 1). MHPA enabled extremely high depth of coverage, with median read depths before and after UMI consensus compression of 185,935× and 19,942×, respectively, with *TSC2-*MHPA and 217,679× and 35,997×, respectively, with *TP53-*MHPA, for a median UMI compression of 9-fold for *TSC2* and 6-fold for *TP53* (Supplemental Figure 2A). DNA input was generally 10–50 ng for each of the 20-plex (*TSC2*-MHPA)/6-plex (*TP53*-MHPA) reactions. Since 10–50 ng is equivalent to 2000–8000 haploid genomes, our maximum sensitivity is 0.05% to 0.01%.

During optimization of *TSC2*-MHPA and *TP53*-MHPA assays, amplicons were labeled using a fluorescent dye (6-carboxyfluorescein [6-FAM]) followed by capillary electrophoresis to assess uniformity of amplification (Figure 2A, quality control step 3; and Supplemental Figure 2B). A high correlation was seen between capillary electrophoresis peak heights and amplicon depth of coverage (*TSC2*-MHPA read depth before and after UMI consensus compression: *r* = 0.71, *P <* 0.0001, and *r* = 0.40, *P <* 0.0001), as expected, indicating that this was a robust method to predict read depth, and enable optimization of the MHPA primer setup (Figure 2A and Supplemental Figure 2C).

Read depth in the *TSC2-* and *TP53*-MHPA assays was relatively uniform across all amplicons, with a maximum fold change (comparing median coverage of the amplicons with highest and lowest read depth) of 4.6 for *TSC2-*MHPA and 4.2 for *TP53-*MHPA (Supplemental Figure 2D). As expected, UMI consensus compression caused a greater reduction in sequencing depth for lower amounts of input DNA for MHPA (for *TSC2*-MHPA, *r* = 0.63, *P <* 0.0001; for *TP53*-MHPA, *r* = 0.65, *P <* 0.0001) (Supplemental Figure 2E).

Forty-seven of 81 samples included in this study were analyzed previously in our laboratory using hybrid capture (MPS of the entire exonic and intronic extent of *TSC1* and *TSC2*; VAF limit of detection: ~0.5%) and/or amplicon MPS (high–read depth targeted validation of the findings from hybrid-capture MPS) (11, 26). These samples were derived from individuals with TSC with low-level systemic *TSC2* mosaicism detected previously in various fluids and tissues, including blood, saliva, semen, normal skin, and TSC skin lesions, available from each patient (including 19 FAF, 10 TSC normal skin, 13 blood, 1 semen, and 1 UF samples) or heterozygous *TSC2* mutations (3 TSC nipple angiofibroma [AF] samples) (Table 1 and Supplemental Table 2). Thirty-five of 36 findings (97%) identified in our previous MPS analyses were validated by our *TSC2*-MHPA assay, with very high positive correlation of the observed VAFs (*r* = 0.99, *P <* 0.0001), confirming the reliability of MHPA (Supplemental Figure 3). The only finding that was not validated had been identified in a FAF, and also seen in a matched blood sample at low VAF (0.17%); the variant was not validated in MHPA analysis of the blood sample, suggesting that the earlier finding had been overcalled. In addition, MHPA done independently twice on the same sample for 8 samples showed extremely high concordance for mutation detection, 100% for VAF ≥ 0.08% (*r* = 0.99, *P <* 0.0001; Supplemental Table 3 and Supplemental Figure 4). At a VAF lower than 0.08%, many but not all variants were seen in both samples, consistent with random effects on inclusion/amplification of a variant allele when it occurs at very low VAF.

MHPA provides at least a 10-fold improvement in sensitivity (detection of variants with VAF 0.01%–0.05%) in comparison with our previous MPS approach for TSC mutation detection (11, 12).

### Results of MHPA analysis of TSC FAFs and other TSC samples.

MHPA was used for the analysis of several different types of samples: (a) TSC facial angiofibroma biopsies (“TSC-FAF”); (b) TSC nipple angiofibroma biopsies (“TSC-nipple AF”); (c) TSC normal skin, mostly from upper arm (“TSC-NS”); (d) samples from TSC blood, semen, and buccal swab (“TSC-blood/semen/buccal swab”); (e) TSC ungual fibroma biopsy (“TSC-UF”); (f) normal skin samples from individuals without TSC, which were adjacent to resected basal cell carcinoma (BCC) lesions on different sun-exposed body areas (“nonTSC-BCCadj NS”); (g) normal skin samples from individuals without TSC, from inner upper lateral arm (reduced sun exposure) (“nonTSC-NS”); and (h) neonatal foreskin samples from individuals without TSC (no sun exposure) (“nonTSC-foreskin”) (Table 1 and Supplemental Table 4). Note that 9 of 13 TSC blood and 6 of 10 TSC normal skin samples were matched (derived from the same TSC individual) with the respective samples from the TSC-FAF set.

### Shower of UV-related TSC2 mutations within FAFs, defining thousands of subclinical “micro-FAFs” in TSC facial skin.

**Figure 3:**
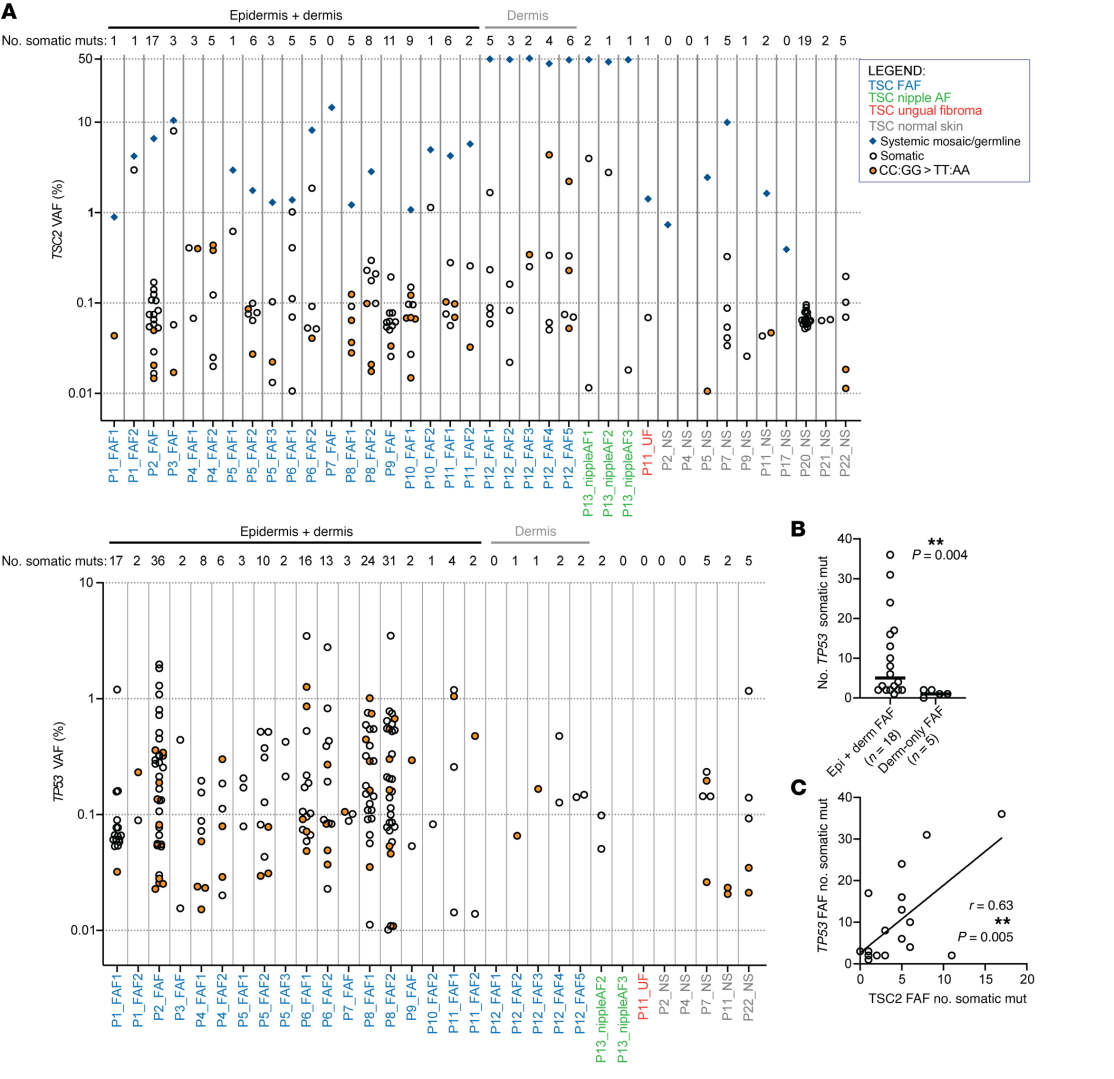
Summary of *TSC2* and *TP53* mutations identified using MHPA in TSC skin samples. (**A**) Top: *TSC2*. Bottom: *TP53*. Blue diamonds indicate systemic mosaic or heterozygous germline (VAF = 50%) mutations, while circles indicate somatic mutations; orange-filled circles correspond to CC:GG>TT:AA mutations. The *y* axis indicates the VAFs, while the *x* axis indicates sample labels; the colors of sample labels correspond to different TSC sample subgroups, as indicated in the inset legend. No. somatic muts: number of somatic mutations observed in each of the samples; Epidermis+dermis: whole-skin biopsies; Dermis: biopsies with removed epidermis. (**B**) Comparison of the number of somatic *TP53* mutations in FAF whole-skin biopsies (Epi + derm FAF) and FAF biopsies with removed epidermis (Derm-only FAF). The comparison was performed using Mann-Whitney test. The horizontal bars indicate median values. **P < 0.01. (**C**) Correlation analysis between the number of somatic mutations in *TSC2* and that in *TP53* in the respective whole-skin biopsies; *r* represents Pearson’s correlation coefficient. The curve was generated using linear regression.

*TSC2*-MHPA analysis of 24 FAFs (TSC-FAF set) confirmed all 20 variants identified in 19 of the 24 FAFs by our previous analyses, and identified 108 new, and a total of 112 low-VAF (0.01%–8.02%, median 0.08%), somatic single-nucleotide variants (SNVs), dinucleotide variants (DNVs), and indels (Figure 3). None of these somatic *TSC2* mutations seen in FAF were seen in blood (*n =* 9) or normal skin samples (*n =* 6) from the same patient, analyzed by *TSC2*-MHPA (Supplemental Table 2.1).

Since the majority of these new low-VAF somatic variants were functionally inactivating (see below), we hypothesized that they reflected the occurrence of additional subclinical clonal fibroblast populations in these facial biopsies, that could be considered micro-FAF tumors, and set out to examine this hypothesis. The VAF of the *TSC2* systemic mosaic/germline variant was higher than any of the somatic *TSC2* mutation VAFs in each FAF (Figure 3A). In addition, when multiple FAFs derived from a single person were studied (*n =* 8 subjects with ≥2 FAFs analyzed), each FAF had a different spectrum of low-VAF *TSC2* mutations, indicating they had arisen independently, likely in different fibroblast clones (Supplemental Table 2.1).

**Figure 4:**
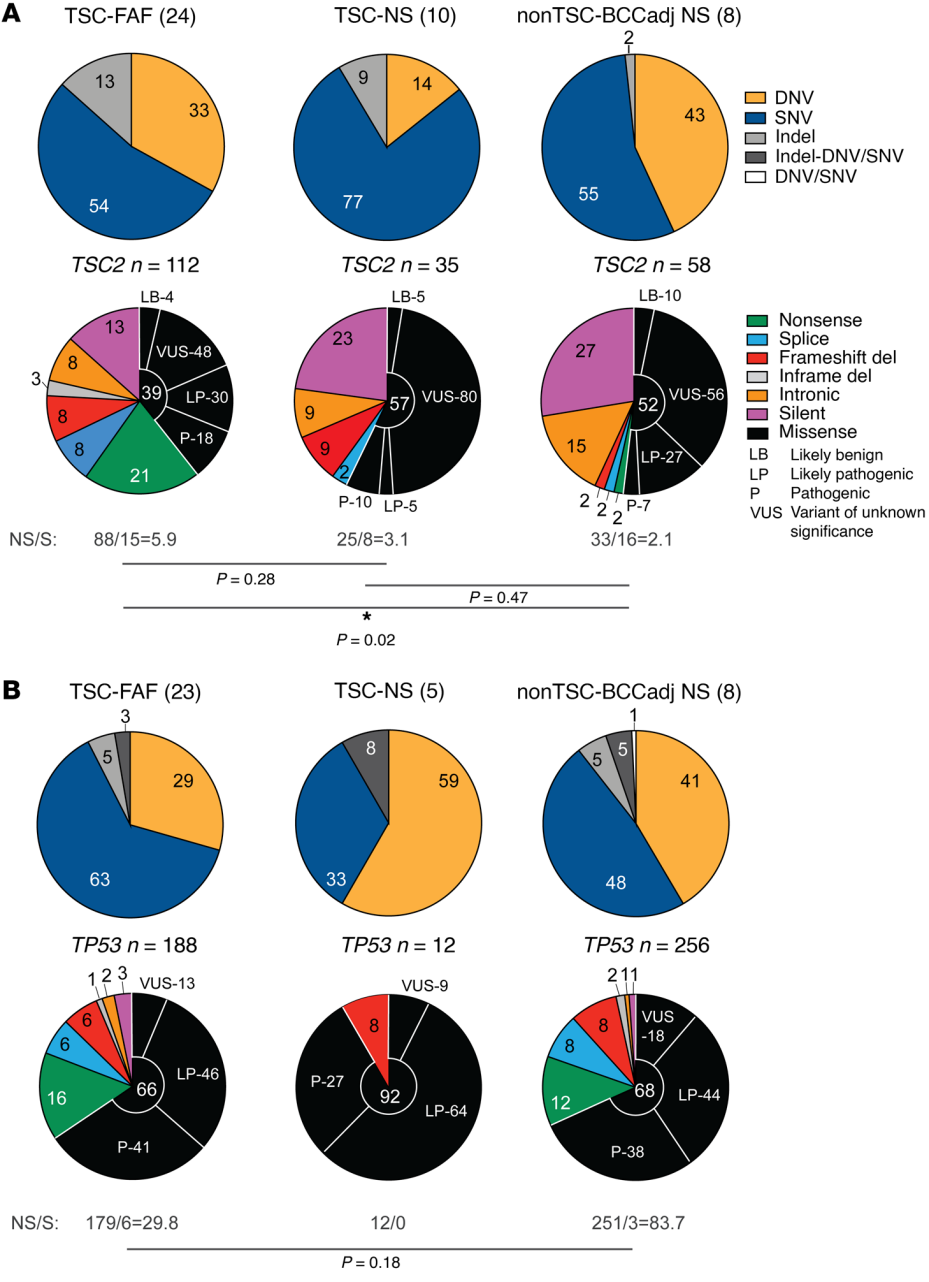
Spectrum of somatic *TSC2* and *TP53* mutations identified using MHPA in skin samples. (**A** and **B**) Summary of somatic *TSC2* (**A**) and *TP53* (**B**) mutations. The number of samples analyzed is indicated in parentheses next to the label for each subgroup of samples (TSC-FAF, TSC-NS, and nonTSC-BCCadj NS); *n* indicates the number of mutations identified in each subgroup. The top pie charts indicate proportions of the identified DNVs, SNVs, and indels. The bottom pie charts indicate proportions of different mutation subtypes color-coded according to the inset legend at left. For missense variants, the functional significance is provided according to the consensus assessment included in Supplemental Tables 2 and 5. *P* values are based on Fisher’s exact test for comparison of nonsynonymous and synonymous variant fractions in the respective subgroups of samples. NS/S, ratio of the number of coding nonsynonymous to synonymous variants. *P < 0.05.

Among the 112 somatic *TSC2* mutations identified in the TSC-FAF set, there were 60 (54%) SNVs, 37 (33%) DNVs, and 15 (13%) indels (Figure 4A). Thirty-four of 37 (92%) DNVs were CC:GG>TT:AA, indicative of UV causation. Fifty-one of 60 (85%) SNVs were also very likely due to UV irradiation based on prior studies (15), with 34 C:G>T:A and 17 G:C>T:A. Fourteen of 15 (93%) somatic *TSC2* indels in TSC-FAFs were deletions, of size 1–18 nt; 1 somatic small *TSC2* insertion was also identified (Supplemental Table 2.1).

Most of the identified somatic non-indel *TSC2* mutations were missense (44 of 97, 45%) or nonsense (23 of 97, 24%) (Figure 4A). Twenty-one of 44 missense variants (48%) were either likely pathogenic or pathogenic, while 21 (48%) were variants of unknown significance (VUS), and 2 (4%) were likely benign (Supplemental Table 2.1 and Figure 4A; and see Methods). The ratio of nonsynonymous to synonymous (NS/S) *TSC2* variants was 5.9 in TSC-FAF, suggesting that they were not background “noise” or passenger events, but rather were inactivating mutations in *TSC2* for the most part, fitting our hypothesis that these low-VAF mutations were driving clonal proliferation of micro-FAFs present in these biopsies.

Further evidence that these mutations were functional were the differences observed in mutation frequency and pattern in TSC-FAF versus TSC-NS and other samples. The NS/S ratio was higher in TSC-FAF (5.9) than in TSC-NS (3.1) and nonTSC-BCCadj NS (2.1) (Figure 4A), suggesting that *TSC2* mutations in nonTSC-BCCadj NS may in many cases be passenger events in keratinocyte clones whose growth is driven by *TP53* and other mutations, while, in TSC-NS, some *TSC2* mutations may be functional and others not.

**Figure 5:**
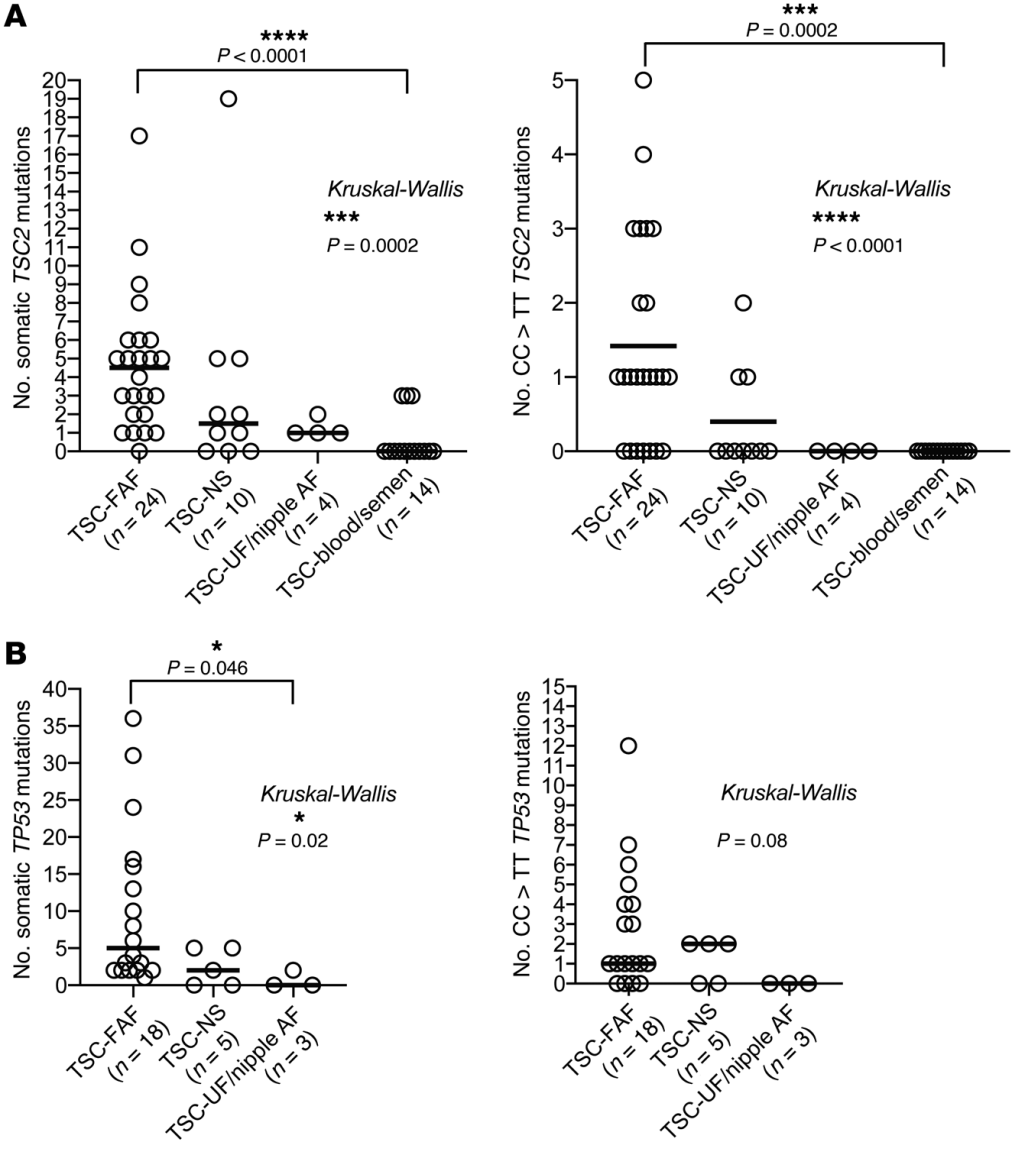
Comparison of the number of mutations identified by MHPA in different groups of TSC samples. (**A**) Left: Each dot represents the number of all somatic *TSC2* mutations in an analyzed sample. Right: Each dot represents the number of all CC:GG>TT:AA *TSC2* mutations in an analyzed sample. (**B**) Left: Each dot represents the number of all somatic *TP53* mutations in an analyzed whole-skin biopsy sample. Right: Each dot represents the number of all CC:GG>TT:AA *TP53* mutations in an analyzed whole-skin biopsy sample. The comparisons were performed using Kruskal-Wallis test. *P* values for the pairwise comparisons within multiple groups were adjusted for multiple comparisons using post hoc Dunn’s test, performed along with Kruskal-Wallis test. Significant Dunn’s *P* values (<0.05) are indicated above the respective plots. The horizontal bars indicate median values. *P < 0.05, ***P < 0.001, ****P < 0.0001.

In addition, the number of all *TSC2* somatic mutations per sample analyzed and the number of *TSC2* CC:GG>TT:AA mutations per sample analyzed were higher in TSC-FAF than in TSC-NS samples (*n =* 10), and higher in TSC-NS than in TSC-UF/TSC-nipple AF (*n =* 4) and TSC-blood/semen samples (*n =* 14) (*P =* 0.0002 and *P <* 0.0001, Kruskal-Wallis test; Figure 5A). At least one CC:GG>TT:AA mutation in *TSC2* was seen in 17 of 24 TSC-FAFs (71%), while no such mutations were observed in TSC-blood/semen/buccal, TSC-UF, or TSC-nipple AF samples (0 of 19, *P <* 0.0001, Fisher’s exact test) (Figure 5A). These findings further confirm the important role of UV radiation in these mutational events occurring predominantly in TSC facial skin, in contrast to other sites.

**Figure 6:**
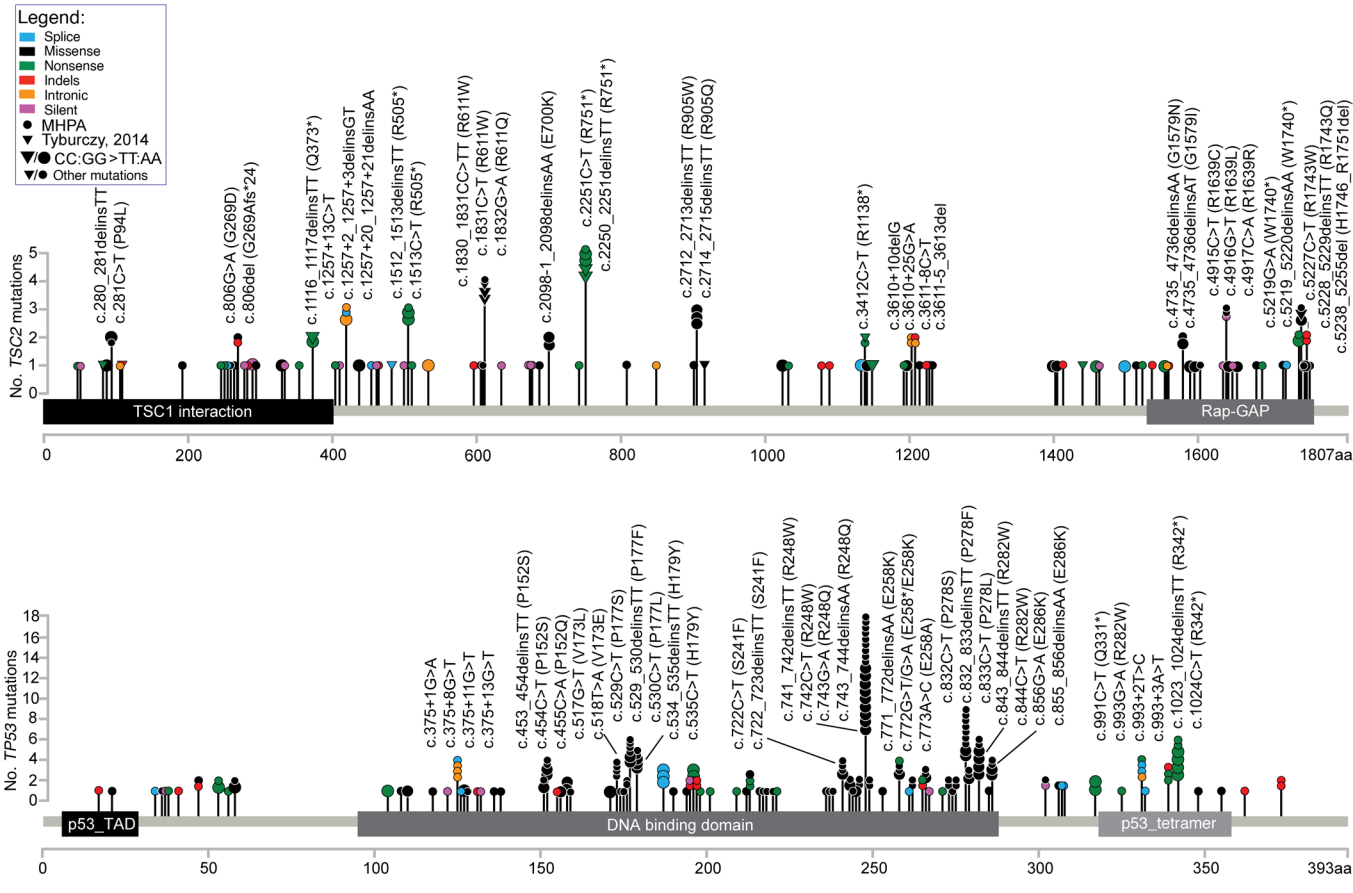
Map of somatic *TSC2* and *TP53* mutations in TSC FAFs. Top: *TSC2*. Bottom: *TP53*. Each lollipop indicates mutation at the amino acid (aa) position indicated below the plot. Circles indicate mutations identified by MHPA, while triangles indicate mutations identified in the MPS study of FAF fibroblast cell cultures performed previously in our group (Tyburczy et al., 2014, ref. 14). The number of circles/triangles and corresponding height of the lollipop correspond to the number of mutations observed at each aa position. Mutations seen at least 4 times are labeled with the nucleotide (c.) and amino acid (p.) position. Types of mutations are color-coded as indicated in the inset legend. Larger symbols correspond to CC:GG>TT:AA UV-related mutations, while smaller symbols indicate all other mutation subtypes.

The distribution of nonsynonymous/intronic somatic mutations identified in TSC-FAFs among the exons of *TSC2* was similar to the distribution of pathogenic germline mutations reported before in the LOVD database (*r* = 0.66, *P <* 0.0001; Supplemental Figure 5A and Supplemental Table 1.1). Most FAF somatic variants were observed just once, but some *TSC2* aa positions appeared to be relative hotspots (Figure 6). Five of the mutated aa positions had been observed to be affected by CC:GG>TT:AA mutation in our previous study of FAF fibroblast cultures (Figure 6 and ref. 14). Thirty of 522 (6%) CC:GG sites in the *TSC2* region sequenced using *TSC2*-MHPA were affected by CC:GG>TT:AA mutation in 1 or more FAFs (Supplemental Table 2.1).

In 11 instances in 7 FAFs, 2 somatic *TSC2* mutations or a somatic and a germline *TSC2* mutation were located in the same amplicon. Scrutiny of the reads using Integrative Genomics Viewer (IGV) indicated that all mutations occurred in *trans*, affecting different alleles (Supplemental Figure 6). In one FAF (P2_FAF) there were 6 somatic low-VAF indels and SNVs in *trans* (VAF 0.017%–0.14%) in the same *TSC2* exon (Supplemental Figure 6).

**Figure 7:**
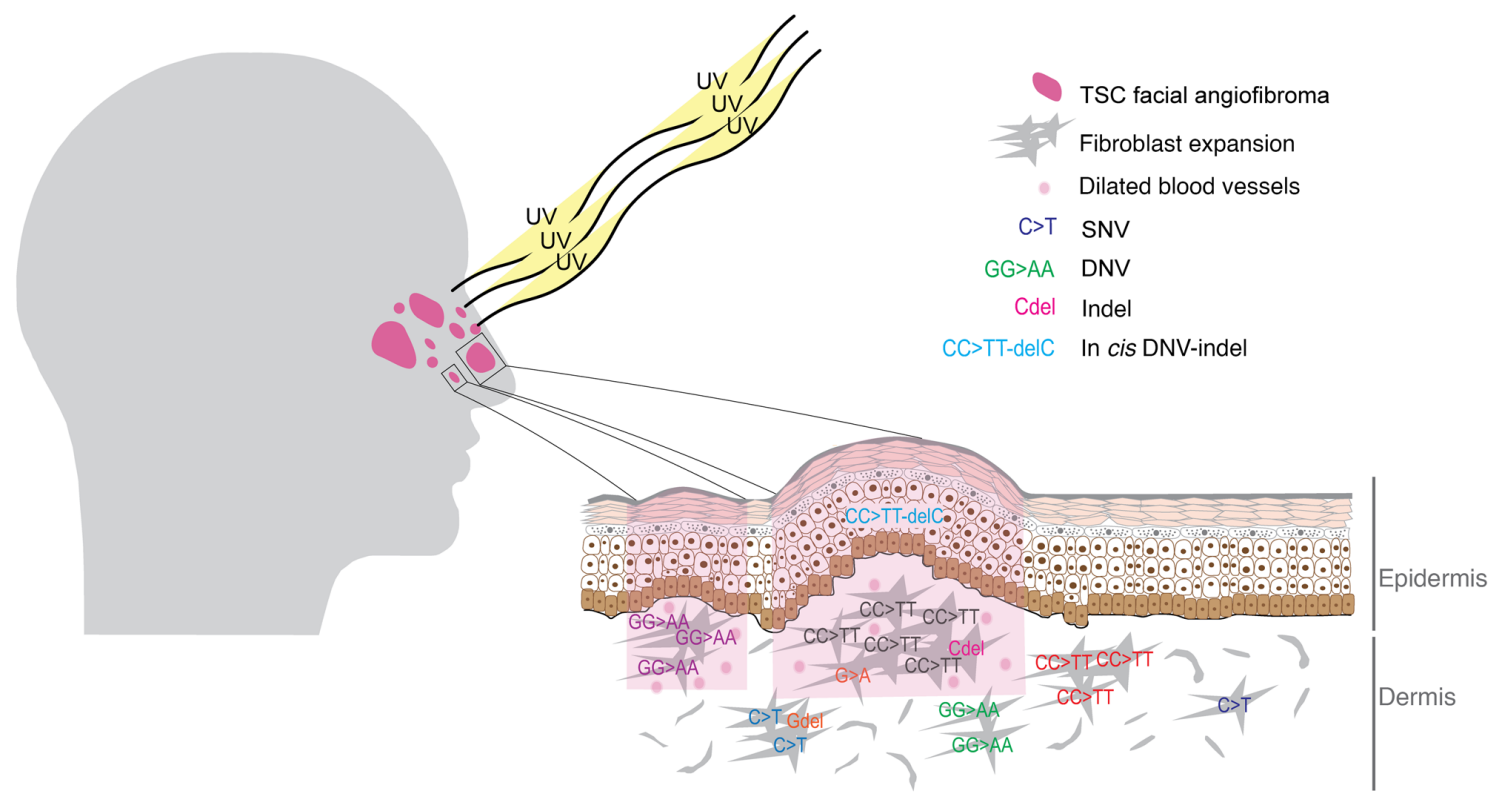
Diagram of UV effects on TSC FAF development. Our findings suggest that thousands of independent clonal fibroblast proliferations (subclinical micro-FAFs) due to second-hit mutations in *TSC2* occur in the skin of TSC2 patients, a small proportion of which develop into observable FAF lesions.

Considering the number of mutations identified, the mosaic/germline VAF in these biopsies, and the size of the total facial skin (see Methods), we estimate that approximately 150,000 independent clonal fibroblast proliferations due to second-hit mutations in *TSC2* occur in the skin of *TSC2* patients (micro-FAFs), a small proportion of which develop into observable FAF lesions (Figure 7). As our MHPA strategy did not assess all exons of *TSC2*, and LOH events are not detected by our methods, this figure may be considered a minimal estimate, and the true number of micro-FAFs may approach 500,000 to 1,000,000 per TSC patient.

We observed that single, clinically visible FAF biopsy contained on average approximately 5 second-hit mutations. Each of the identified second hits may represent either the clinically visible FAF or micro-FAFs nearby. For many of the mosaic TSC-FAFs analyzed in this study (e.g., P1_FAF2, P3_FAF, and P6_FAF1; Supplemental Table 2.1), we were able to identify 1 second-hit mutation with VAF close to the VAF of the systemic mosaic variant, which we think is likely derived from the clinically visible FAF. The multiple additional somatic mutations seen in most samples had significantly lower VAF (0.01%–0.5%), and they are likely derived from other FAF lesions in the specimen (micro-FAF) that were subclinical.

### Validation of new MHPA TSC2 findings by prior MPS.

Forty-one of 110 *TSC2* somatic variants newly identified by MHPA in TSC-FAF or TSC-nipple AF samples were compared with prior hybrid-capture MPS data (mean depth of coverage: ~500×) (11, 26). Ten of 41 (24%) variants with median 0.12% VAF were seen in 1–3 reads, consistent with the MHPA findings (Supplemental Figure 7A). The median VAF of the remaining 31 of 41 (76%) MHPA variants (0.07%) was significantly lower (*P =* 0.002, Mann-Whitney test), explaining their absence in our prior hybrid-capture MPS analysis (Supplemental Figure 7B).

### Comparison of TP53 mutations with TSC2 mutations in TSC-FAF.

*TP53*-MHPA analysis of the TSC-FAF set (Table 1) led to the identification of 188 low-VAF (0.01%–3.50%, median 0.13%) variants: 119 (63%) SNVs, 55 (29%) DNVs, 9 (5%) indels, and 5 (3%) adjacent indel-SNVs (see below for further discussion of the last category). Fifty-two of 55 (95%) DNVs were CC:GG>TT:AA, due to UV mutagenesis. *TP53* SNVs in FAFs were predominantly C:G>T:A (*n =* 63 of 119, 53%) and G:C>T:A (*n =* 36 of 119, 30%), similar to *TSC2* SNVs in FAFs. All 9 *TP53* indels in FAFs were deletions, also similar to *TSC2* indels in FAFs (all but one deletions) (Supplemental Table 5.1).

Most of the non-indel *TP53* mutations in FAF were missense (123 of 174, 71%), the vast majority of which, 107 of 123 (87%), were reported to be likely pathogenic or pathogenic (Figure 4B and Supplemental Table 5.1). The NS/S ratio for *TP53* mutations was extremely high in TSC-FAF (29.8), indicating that *TP53* mutations are under strong selective pressure in TSC facial skin (Figure 4B).

*TP53* mutations were much more common in whole-skin FAF biopsies (dermis + epidermis; *n =* 18, range 1–36 mutations, median 5 mutations) than in FAF biopsies that were dermis only (*n =* 5, range 0–2 mutations, median 1 mutation) (*P =* 0.004, Mann-Whitney test; Figure 3B), suggesting that *TP53* mutations were occurring mainly in keratinocytes in these samples, rather than fibroblasts in TSC facial skin, consistent with previous studies in individuals without TSC (15, 18). In addition, the VAF of somatic *TP53* mutations was significantly higher than the *TSC2* VAF (median VAFs: 0.13 and 0.07, respectively; *P =* 0.0001, Mann-Whitney test) in whole-skin TSC-FAF (Supplemental Figure 8), also suggesting a different cell of origin for the *TP53* mutations. An average of 10.1 (range 1–36) *TP53* mutations were identified per whole-skin FAF, and correlated with the number of *TSC2* mutations in the same sample (*n =* 18, *r* = 0.63, *P =* 0.005; Figure 3C), likely reflecting the role of UV irradiation in the generation of both genes’ mutations, albeit in different cell types.

The number of *TP53* mutations was significantly higher in whole-skin TSC-FAF (median 5) than in TSC-NS (median 2) and TSC-UF/TSC-nipple AF (median 0) samples (Kruskal-Wallis test, *P =* 0.02), owing to expected differences in UV exposure in these biopsy sites (Figure 5B). No CC:GG>TT:AA *TP53* mutations were seen in TSC-nipple AF or TSC-UF samples, in contrast to whole-skin TSC-FAF, where 14 of 18 (78%) biopsies had at least one CC>TT mutation, similar to our findings for UV-induced mutations in *TSC2* in these samples.

The distribution of the nonsynonymous/intronic somatic *TP53* mutations in FAFs mirrors the distribution of somatic mutations reported previously in normal keratinocytes/skin cancers (15–17, 27–39) (*r* = 0.92, *P <* 0.0001; Supplemental Figure 5B and Supplemental Table 1.2), including multiple mutations at well-known hotspots (e.g., aa 248) (Figure 6). Twenty-seven of 175 (15.4%) CC:GG sites in the region targeted by *TP53*-MHPA were affected by CC:GG>TT:AA mutation in 1 or more FAFs (Figure 6 and Supplemental Table 5.1).

### TSC2 and TP53 mutations in normal-appearing skin.

*TSC2*-MHPA and *TP53*-MHPA analysis of 8 samples from sun-exposed nonTSC-NS adjacent to BCC revealed a large number of somatic *TSC2* and *TP53* mutations (7.3 and 32.0 mutations per sample, respectively) (Supplemental Figure 9). A large fraction of the mutations were CC:GG>TT:AA for both genes (*TSC2*: 22 of 58 [38%]; *TP53*: 101 of 256 [39%]) (Supplemental Figure 9A and Supplemental Tables 2.2 and 5.2).

We also performed *TSC2*-MHPA analysis of 2 additional panels of normal skin biopsies from individuals without TSC: (a) a panel of 10 normal skin biopsies from the inner upper lateral arm (area with reduced sun exposure) (nonTSC-NS) and (b) a panel of 10 biopsies from newborn foreskin (nonTSC-foreskin). In contrast to the sun-exposed nonTSC-NS, these samples had an average of 0.7 and 0.5 *TSC2* variants, respectively, with a very low VAF (median 0.09%) (Supplemental Figure 9 and Supplemental Table 2.2). As expected, none of the nonTSC-foreskin DNA samples showed a CC:GG>TT:AA mutation, and just a single such mutation was seen in the sun-protected nonTSC-NS.

### UV-induced-mutation signatures in TSC-FAF and normal skin.

**Figure 8:**
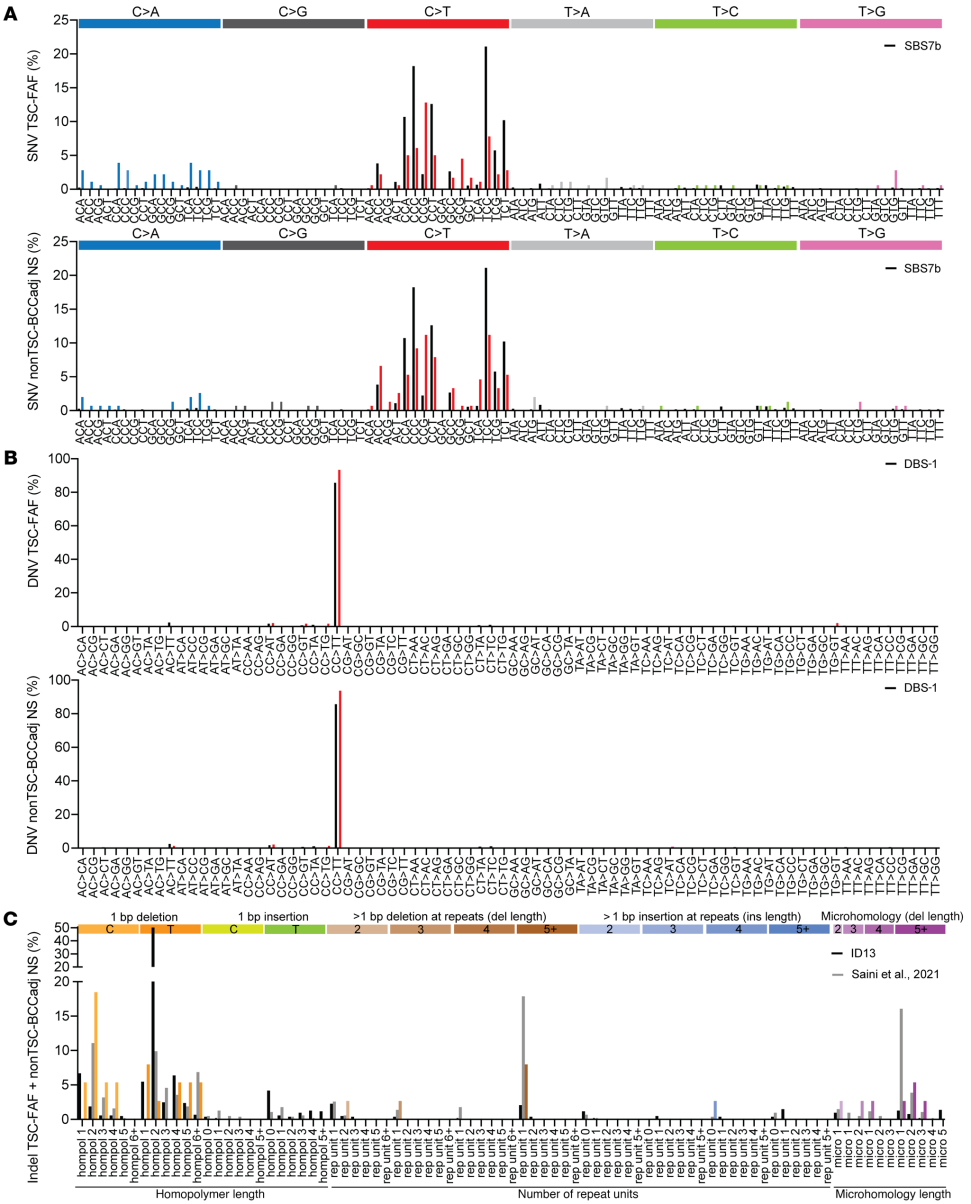
Mutation signatures in TSC-FAFs and nonTSC-BCCadj normal skin samples. (**A**) Comparison of the reference SNV COSMIC UV-induced-mutation signature SBS7b (indicated in black) and the SNV mutation signature identified using MHPA (colors of the bars correspond to the colors of different single-nucleotide substitutions indicated above the plot). The signatures are summarized separately for TSC-FAFs (top plot) and nonTSC-BCCadj normal skin (bottom plot). (**B**) Comparison of the reference DNV COSMIC UV-induced-mutation signature DBS-1 (indicated in black) and the DNV mutation signature identified using MHPA (indicated in red) in TSC-FAFs (top plot) and nonTSC-BCCadj normal skin (bottom plot). (**C**) Comparison of the reference indel COSMIC UV signature ID13 (indicated in black), the indel UV signature by Saini et al. (42) (indicated in gray), and the indel signature identified using MHPA (colors of the bars correspond to colors of different single-nucleotide substitutions indicated above the plot). The indel signature summarizes combined MHPA results for TSC-FAFs and nonTSC-BCCadj normal skin.

We combined all somatic *TSC2* and *TP53* mutations from all samples in the respective TSC-FAF and nonTSC-BCCadj NS sets, to enable comparison with canonical mutation signatures from the Catalogue of Somatic Mutations in Cancer (COSMIC) (refs. 40–42 and Figure 8, A–C). The SNV signatures for the TSC-FAF and nonTSC-BCCadj NS sets showed the highest cosine similarity to UV-induced-mutation signature SBS7b, which has a predominance of C>T substitutions, with cosine similarity scores 0.65 and 0.83, respectively (Figure 8A and ref. 40). However, there were larger numbers of C>T substitutions in the CCG and GCG contexts in TSC-FAF, and in the CCG context in nonTSC-BCCadj NS (Figure 8A). A modest enrichment for G:C>T:A substitutions in variable sequence contexts was also noted in the TSC-FAF set, less so for nonTSC-BCCadj NS. This may be due to ROS generated by sunlight, as reported previously (Figure 8A and ref. 15).

**Figure 9:**
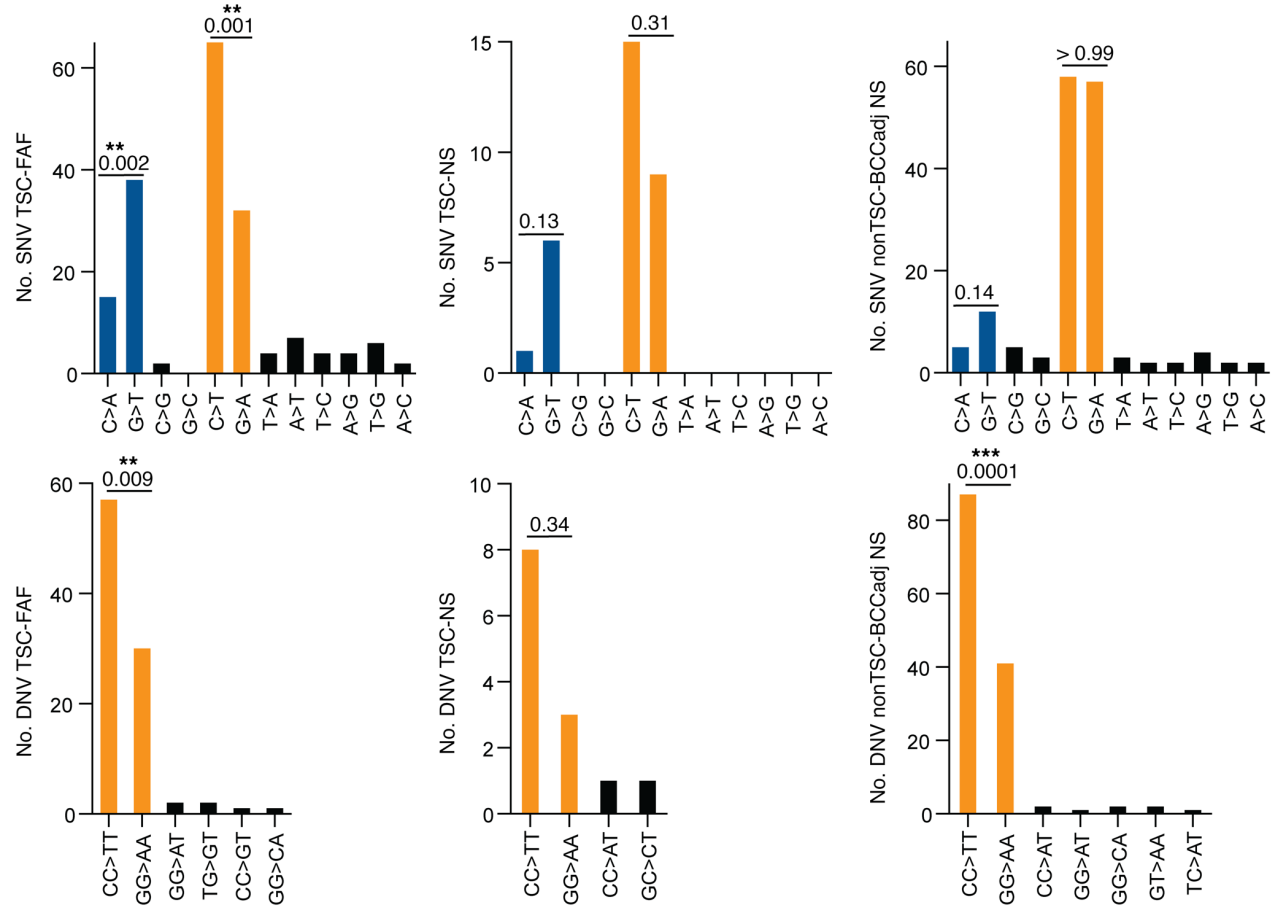
Orientation of SNVs and DNVs relative to transcription. Total number of *TSC2* and *TP53* mutations in the coding (untranscribed) versus the noncoding (transcribed) strand for all detected SNVs (top row) and DNVs (bottom row). The labels for different subgroups of the analyzed samples (TSC-FAF, TSC-NS, and nonTSC-BCCadj NS) are given next to the *y* axis. *P* values are indicated for untranscribed/transcribed strand bias, for C>A/G>T, C>T/G>A, and CC>TT/GG>AA (binomial distribution test), above each of the compared pairs. **P < 0.01; ***P < 0.001.

DNV signatures for both TSC-FAF and nonTSC-BCCadj NS each matched very well with the canonical COSMIC DBS-1 UV signature, with identical cosine similarity scores of 0.999 (40), with CC:GG>TT:AA substitutions accounting for 85.8%, 93.5%, and 93.9% of all DNV substitutions in the DBS-1, TSC-FAF, and nonTSC-BCCadj NS, respectively (Figure 8B). For each C:G>T:A SNV and CC:GG>TT:AA DNV, more C than G (TSC-FAF: *P =* 0.001, binomial distribution test) and more CC than GG (TSC-FAF: *P =* 0.009, binomial distribution test) were mutated, consistent with an untranscribed strand bias resulting from transcription-coupled nucleotide excision repair. A strand bias for G:C>T:A mutations was also noted, with more G than C mutated (TSC-FAF: *P =* 0.002, binomial distribution test), as reported previously for UV-exposed eyelid epidermis (Figure 9 and ref. 15).

The indel signature observed for all indels identified in TSC-FAF and nonTSC-BCCadj NS skin samples combined (*n =* 38 indels) was different from the reference COSMIC UV-related ID13 signature, with a cosine similarity score of 0.24 (Figure 8C). It was more similar to the recently reported indel signature identified from whole-genome MPS of single-cell-derived clonal lineages from primary normal skin cells (42), with a cosine similarity score of 0.46. Similar to the findings of Saini et al. (42), most single-nucleotide deletions, 23 of 38 (61%), occurred in a 2- to 5-nt poly-C:T/G:A homopolymer tract. Deletions with microhomology at the breakpoints were also identified, accounting for 9 of 38 indels (24%).

### A recurrent complex mutation type in TP53 only.

**Figure 10:**
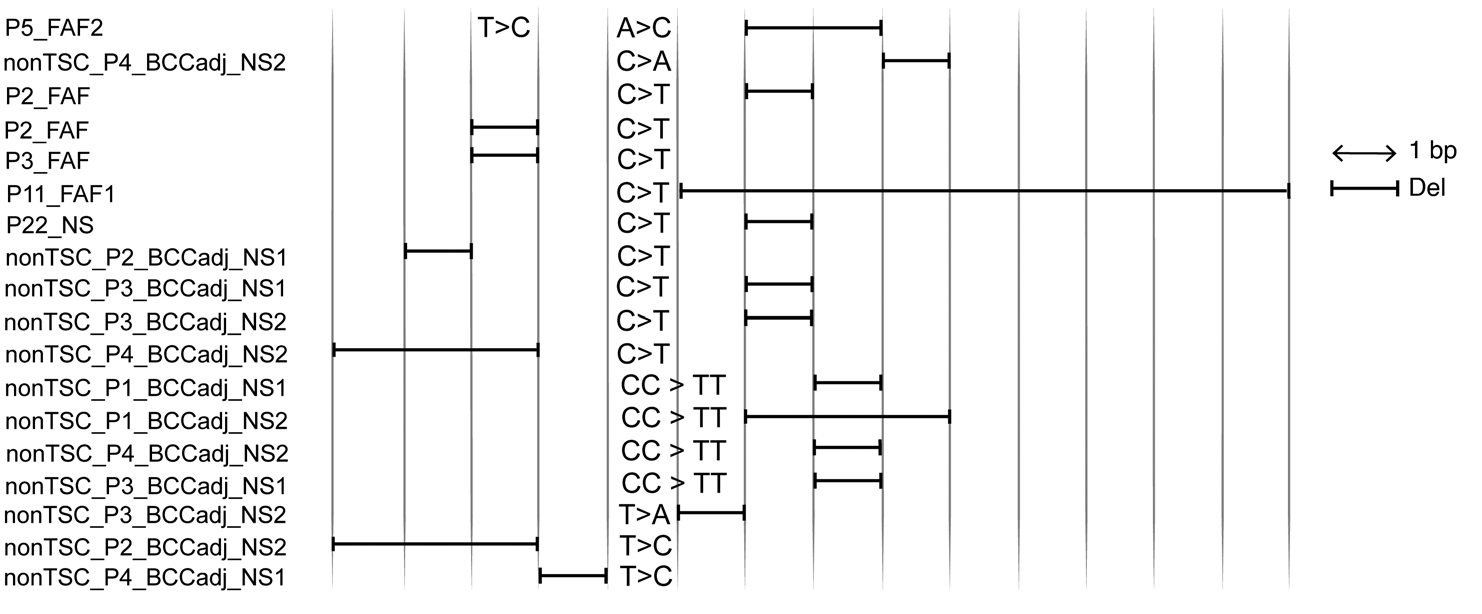
A complex mutation type in *TP53* only. Diagram of the adjacent indels and SNVs/DNVs, occurring in *cis*. Column width is 1 nt. UV-related SNVs/DNVs are aligned according to the primary UV-related cyclobutyl pyrimidine dimers reflected by C>T/CC>TT DNA damage.

Interestingly, 18 complex adjacent indel-SNV/DNV mutations in *TP53* were identified, likely generated by UV radiation, with 12 in nonTSC-BCCadj NS, 5 in TSC-FAF, and 1 in TSC-NS (Figure 10 and Supplemental Figure 10). These adjacent indel-SNV/DNV mutations were observed in *TP53* only (*TP53*: 18 of 455 somatic mutations in skin; *TSC2*: 0 of 210 somatic mutations in skin; *P =* 0.001, Fisher’s exact test), which suggests that these mutations occur in keratinocytes only and may be due to effects of UVB radiation, which does not reach the dermis (43, 44). Eight of 18 (44%) indel-SNV/DNV mutations occurred within a tract of at least 3 adjacent Cs or Gs (Supplemental Figure 10). Thirteen of 18 (72%) indel-SNV/DNVs contained either C:G>T:A or CC:GG>TT:AA UV-related substitutions; for 9 of these 13 (69%) the UV-related SNV/DNV was upstream of the deletion, while for 4 it was downstream of the deletion (Figure 10).

### Somatic mutation prevalence and clinical characteristics of skin biopsies and their donors.

We examined the possibility that the prevalence of somatic *TSC2*/*TP53* mutations in skin biopsies might be associated with different clinical characteristics, including FAF grade (Facial Angiofibroma Severity Index score; ref. 45), age at biopsy, pigmentation, latitude at which donors had lived for most of their lives, and degree of sun exposure. We did not find a strong correlation between most of these factors and number of somatic mutations (Supplemental Figure 11), likely owing to complex interactions among these factors in determining mutation occurrence. However, there was a trend toward fewer somatic mutations in either *TSC2* (*P =* 0.03, Kruskal-Wallis test) or *TP53* in skin types with higher amounts of pigment (Supplemental Figure 11D). There was also a significant association between the number of either *TSC2* (*P <* 0.0001, Kruskal-Wallis test) or *TP53* (*P =* 0.02, Kruskal-Wallis test) somatic mutations and degree of sun exposure at the specific sites of different skin biopsies (Supplemental Figure 11E).

## Discussion

TSC hamartomas are notorious for their highly variable age of onset and unpredictable clinical behavior. For example, cardiac rhabdomyomas are present in the majority of TSC infants at birth but spontaneously resolve in most by 5 years of age. FAFs typically begin to appear at age 5–12 years, and continue to progress through approximately age 40 unless effective local or systemic therapy is applied.

The importance of UV sunlight exposure in the pathogenesis of BCC and squamous cell carcinoma (SCC), arising from keratinocytes, and of melanoma, arising from melanocytes, has been known for many years but has been brought into sharper detail by exome and genome sequencing of these tumors (16, 17, 39), as well as MPS analysis of UV-induced mutations in normal epidermis (15, 18, 19, 27, 46). Several years ago, we discovered that UV-induced mutation was also an important factor in the development of TSC FAF, as second-hit mutations in both cultured fibroblasts and FAF biopsies were identified consistent with UV-induced effects (particularly CC>TT). TSC FAFs were to the best of our knowledge the first skin tumor with origin in the dermis to be identified with UV radiation–induced mutations. This finding initiated a major change in the care of individuals with TSC, with provision of advice to avoid facial sun exposure to minimize risk of FAF development at all ages.

Although standard MPS has been used for mutation identification in many studies, there is a lower limit on the VAF, approximately 0.5%, that is detectable using non-UMI MPS methods owing to the intrinsic error rate and “noise” in this process (20–22). Here we have developed a UMI-based MPS strategy, easily adapted to any gene or genomic region of interest, that is capable of VAF detection as low as 0.01%, a 10- to 50-fold improvement in sensitivity in comparison with standard MPS methods for variant detection (15, 18, 19, 27, 46).

Our study revealed that somatic mutations in *TSC2* and *TP53* are a pervasive phenomenon in TSC-FAF biopsies (4.8 and 10.1 mutations per whole-skin biopsy, respectively), including those from individuals mosaic for a *TSC2* mutation at low allele frequency (Figure 3A). Our data fit the model that additional *TSC2* mutations occur in small clonal populations of dermal fibroblasts that are undergoing clonal expansion but are not recognized clinically; these clonal populations represent a tumor that we call a micro-FAF. Unexpectedly, even higher somatic mutation rates were observed in both *TSC2* and *TP53* (7.3 and 32.0 mutations per sample, respectively) in nonTSC-BCCadj NS biopsies from sun-exposed normal skin adjacent to BCC lesions (Supplemental Figure 9). However, several factors likely contribute to the high rate of mutation in those samples: (a) the nonTSC-BCCadj NS biopsies were derived from older individuals (range 51–75 years, median 59) in comparison with TSC-FAF biopsy individuals (range 15–52 years, median 32) (*P =* 0.002, Mann-Whitney test); (b) these NS biopsies were likely highly exposed to UV radiation, which is the primary risk factor for BCC; and (c) the median *TSC2* mosaic allele frequency in the TSC-FAFs was quite low (3.6%), reflecting a smaller proportion of “at-risk” fibroblasts for clonal outgrowth following a second *TSC2* mutation. In addition, in TSC-FAF and nonTSC-BCCadj NS, the nonsynonymous mutation rate per whole-skin FAF biopsy sample was 3-fold and 8-fold higher, respectively, in *TP53* than in *TSC2*, which is similar to the 4- to 5-fold ratio seen in BCC and SCC derived from keratinocytes (16, 38, 39) and melanoma (cBioPortal; refs. 17, 47, 48). These observations suggest that in keratinocytes, *TSC2* mutations may be passenger events, occurring at random and being carried along in keratinocyte clones whose growth is driven by *TP53* and other mutations (16, 17, 38, 39, 47, 48). In contrast to the appreciable numbers of *TSC2* mutations identified in these studies, targeted MPS of 232 punch (0.25 mm) biopsies of normal epidermis (limit of detection: VAF ~2%) revealed only 2 nonsynonymous mutations in 232 samples analyzed (0.9%), a rate that was 22-fold higher in *TP53* (18). Combined with our observation that *TP53* mutations are depleted in TSC-FAF biopsies with the epidermis removed, these findings support the model that *TSC2* mutations in TSC FAF samples occur mostly in fibroblasts, while *TP53* mutations occur in keratinocytes (Figure 3).

As expected, we observed a significantly lower *TSC2* mutation rate in nonTSC normal skin samples with either reduced sun exposure (nonTSC-NS from upper inner arm) or no sun exposure (nonTSC-foreskin) (0.7 and 0.5 mutations per biopsy, respectively). The incidental *TSC2* mutation findings in these nonTSC normal skin samples may be due to either mild sun exposure for some of the nonTSC-NS samples from arm, or non-UV-related mutagenic processes, including spontaneous cytosine deamination at the CpG sites, oxidative DNA damage, and DNA replication errors, as reported recently (42). It is not possible to define a specific mutation signature/mutation mechanism from our study, since there were so few *TSC2* variants identified in these biopsies with either reduced or no sun exposure. However, several studies have provided some insight into this question, with identification of mosaic mutations at relatively high frequency in normal skin fibroblasts (49), in diverse tissues apparently due to spontaneous deamination of methylated cytosines (50), and occurrence of cancer-related somatic mutations in sun-shielded normal skin (19) and normal esophagus epithelium (51).

In addition, these low-frequency somatic variants in *TSC2* occurring in normal skin (and likely other tissues) may help to explain the occurrence of sporadic FAF and other TSC hallmark tumors in the non-TSC population. Solitary FAFs may occur as single isolated lesions in as much as approximately 8% of the general non-TSC population (52). Sporadic kidney angiomyolipoma, a hallmark TSC-associated tumor, is also seen in approximately 0.1%–0.2% of the general adult population (53, 54).

SNV mutation signatures were similar for TSC-FAF and nonTSC-BCCadj NS, and matched well with the canonical UV-related signature SBS7b (40). Minor differences were noted, which may relate to the 2 genes (*TSC2* and *TP53*) under analysis here. However, there was a significant enrichment for C>T substitutions in the CCG context in comparison with SBS7b, for both TSC-FAF and nonTSC-BCCadj NS. Enrichment for this same mutation and context was also reported by Wei et al*.* (19), who compared mutation patterns in skin with UV exposure with those seen in non-sun-exposed skin. G:C>T:A mutations were also relatively common in both TSC-FAF and nonTSC-BCCadj NS, as previously reported in sun-exposed skin as a result of heat-related oxidative damage (15, 42, 55).

DNV signatures in both TSC-FAF and nonTSC-BCCadj NS were very similar to the reference DBS-1 DNV signature, where CC:GG>TT:AA constituted more than 85% of the observed DNVs. Interestingly, in our study DNVs constituted a large fraction of all observed mutations (TSC-FAF: *TSC2*, 33%; *TP53*, 29%; nonTSC-BCCadj NS: *TSC2*, 43%; *TP53*, 41%), and these findings were similar to the DNV fraction from our previous study of TSC fibroblast FAF cultures, for which the CC>TT DNV was seen in half of samples (14). However, this mutation type has been identified at much lower levels in previous deep-MPS analyses of normal skin, 3% to 9% (15, 18, 19, 55). We suspect that our findings are accurate, and our detection is enhanced by our specialized computational pipeline for DNV detection, as well as careful visual review of all mutation calls using IGV. Although there are certainly other possible explanations, we suspect that DNVs were likely undercalled in many other reports, since DNVs are not efficiently called and annotated by most of the currently available algorithms for detection of small mutations (56).

We identified a refined UV-related indel signature that is different from the recently proposed ID13 (40). Our signature is dominated by single-nucleotide deletions at homonucleotide repeats, similar to findings from whole-genome MPS of single-cell-derived clonal lineages from primary normal skin cells (42). We also observed a separate class of complex adjacent indels-SNV/DNVs in skin samples, which to the best of our knowledge has not been reported before in MPS studies of human samples.

Our study has some limitations. First, MHPA analysis does not enable one to distinguish whether mutations occur in the same or different clones in most cases. Several examples of mutations occurring in *trans* were identified, from review of sequence data files in IGV, although this does not prove that the mutations were derived from different cells. A second limitation is that our conclusion that the observed *TSC2* mutations are occurring in fibroblasts, rather than (or in addition to) other dermal populations, is largely based on previous fibroblast culture studies (14). Another potential limitation is that the depth of coverage among the MHPA amplicons is somewhat uneven. However, considering the multiplex PCR used for universal amplification of the queried sequences, there were no major differences in coverage of distinct amplicons, with an approximately 4-fold difference in median coverage of amplicons with minimum versus maximum coverage for both *TSC2-* and *TP53*-MHPA assays. Last, our MHPA focused on mutation-rich regions of *TSC2* and *TP53*, covering 74% of all germline *TSC2* and 93% of all somatic *TP53* mutations. It is certain that UV-induced mutations affect the other regions of each gene, not covered in our assays. In addition, MHPA will not detect copy-neutral or copy-loss LOH events in which the wild-type allele of *TSC2* is lost at low allele frequency. Hence, our estimate of roughly 150,000 micro-FAFs occurring in the facial skin of TSC subjects may be considered a minimum estimate.

In summary, UV radiation–induced small point mutations are highly prevalent in the facial skin of TSC subjects, in both *TSC2* and *TP53*. Such *TSC2* mutations are not seen in TSC saliva, blood, and other non-sun-exposed skin samples, suggesting that they are due to UV exposure of facial skin and the positive growth effects of biallelic loss of *TSC2* on facial dermal fibroblasts, which develop into incipient FAF tumors, which we term micro-FAFs. Thus, in assessing the facial skin of TSC individuals, one is looking at the most robust growth of tens to hundreds of thousands of fibroblast clones, whose growth is driven by 2-hit loss of *TSC2* and likely influenced by potential cell-intrinsic and -extrinsic effects, including specific fibroblast subtype and microenvironment. Finally, we identified a recurrent complex mutation signature pattern in *TP53*, consisting of both an SNV or DNV and a deletion.

## Methods

### Experimental design.

In this study, an MHPA ultra-deep sequencing strategy has been developed and applied for the analysis of a total of 81 samples (TSC and non-TSC samples; Table 1 and Figure 1), for characterization of UV-specific mutations in human skin, focusing on TSC facial skin.

### Patient recruitment and sample collection.

Demographic and clinical data were collected for all the TSC subjects, based on medical records and self-reported questionnaire. All subjects had a full clinical examination by a health care provider familiar with TSC.

Circular 2-mm punch biopsies of TSC-FAF were performed by dermatologists to ensure that the dermal component of the angiofibroma was obtained. Each of the collected FAF biopsies was a distinct, single clinically visible lesion. Nineteen FAF biopsies were small lesions and included both dermis and epidermis. Five FAF biopsies were larger lesions and contained dermis only, with the epidermis layer removed by laser treatment. The TSC-UF was collected by shave biopsy/tangential excision. The TSC-NS biopsies were 4- to 6-mm circular punch biopsies (specific TSC-NS biopsy sites are indicated in Supplemental Table 4). The TSC-nipple AFs were 1- to 3-mm biopsies, affecting the nipple and/or areola (26). The nonTSC-BCCadj NS biopsies were normal skin samples from individuals without TSC, and were obtained from a region adjacent to a BCC being resected (specific nonTSC-BCCadj NS biopsy sites are indicated in Supplemental Table 4). The nonTSC-NS biopsies were 4-mm circular punch biopsies from inner upper lateral arm. The nonTSC-foreskin biopsies were 4- to 5-mm-square biopsies from healthy full-term 2- to 5-day-old newborns undergoing elective circumcision. Skin biopsies were subject to immediate DNA extraction without fixation.

### DNA extraction and quantification.

Extraction of genomic DNA from peripheral blood lymphocytes, buccal swab, semen, and skin biopsies was performed using standard methods (Qiagen QIAamp Mini Kit, catalog 51306). DNA quantification was performed by both QUBIT dsDNA HS (Thermo Fisher Scientific) and PicoGreen assays.

### Hybrid-capture and amplicon MPS analysis.

For 44 of 81 samples included in this study, hybrid-capture MPS (Agilent SureSelect platform) and/or amplicon MPS analyses of *TSC1* and *TSC2* were performed (Illumina platform) as a part of previous studies (11, 13, 26). Three of 81 samples were analyzed using these same MPS methods and had not been reported before. SNVs, indels, and large multiexonic deletions were identified using our custom computational pipeline in Unix, Python, and MATLAB (11).

### Multiplex high-sensitivity PCR assay (MHPA).

Our MHPA strategy is derived from prior publications that used an amplicon-based strategy (22–24). As described in Results, we have developed MHPA for the analysis of *TSC2* and *TP53*. Each of the *TSC2*/*TP53*-MHPA sets (sets I and II) were subject to multiplex PCR amplification in separate PCR reactions (Supplemental Table 1 and Supplemental Table 6.1 and 6.2). If possible, the amplicons from the respective sets were designed to be of different lengths (ideally ≥2 nt) to enable separation of the amplicons in the capillary electrophoresis quality control (QC) step, which was applied to ensure uniformity of amplification of each amplicon (Supplemental Table 1 and Supplemental Table 6.1). Capillary electrophoresis (ABI 3730XL sequencer) was performed at the Center for Computational and Integrative Biology DNA Core Facility at Massachusetts General Hospital (Cambridge, Massachusetts, USA).

### Preparation of the MHPA libraries and sequencing.

MHPA consists of PCR amplification of short DNA segments that include UMI barcodes, followed by sequencing (Supplemental Table 6). Each of the *TSC2*/*TP53* pair sets is used to amplify DNA in 3 sequential reactions. In the first reaction, a linear amplification of each genomic region occurs using reverse primers only that include a random 14-nt UMI (“Rev-UMI”). Following SPRIselect bead (Beckman Coulter, B23318) purification, another linear amplification of each genomic region occurs using forward primers only (“Fwd”). Following bead purification, in the third reaction, amplification of the UMI-barcoded molecules occurs using universal primers (“Fwd_P5_UP” + “Rev_index_P7_UP”) (Supplemental Table 6.1). Both linear and PCR amplifications were performed using Q5 High-Fidelity DNA Polymerase (New England Biolabs, M0493L). The MHPA libraries were purified on Select-A-Size DNA Clean & Concentrator columns (Zymo Research, D4080), assessed on agarose gel (1.5%) and Agilent TapeStation as QC, and quantified using QUBIT dsDNA HS assay and quantitative PCR. Sequencing analysis was performed on MiniSeq or NextSeq 500 Illumina sequencers (paired-end 150-nt sequencing) in the Molecular Biology Core Facilities at Dana-Farber Cancer Institute. Sequences of universal primer (UP) index and sequencing primers are included in Supplemental Table 6.3.

### MHPA data processing and computational analysis.

Pooled sequenced samples were demultiplexed and converted to FASTQ files. Fastp software (57) was used for preprocessing of FASTQ files to trim adapter sequences and extract the UMI sequence that is appended to the read name. Reads were mapped to the human reference genome (hg19) using the Burrows-Wheeler Aligner (BWA; v0.7.15). For sequencing error suppression, MHPA read data were compressed to UMI consensus reads using Gencore software (58). For variant identification, consensus reads were generated for UMI families with at least 2 reads, in which at least 95% of reads had the same variant. For reporting the VAF, we counted all UMI families in which more than 50% of reads had the variant. We then analyzed the consensus read data using our custom computational pipeline in Python/MATLAB, derived from the pipeline described previously (11, 12, 59), to identify all SNV, DNV, and indel variants in *TSC2*/*TP53*. DNV identification was performed by manual Integrative Genomics Viewer (IGV) (60) review of all SNV calls occurring in adjacent genomic positions. Variant Effect Predictor (VEP) (61) was used for SNV annotation.

Our in-house Python/MATLAB pipeline scripts are available in the GitHub repository (https://github.com/kklonows/MHPA_pipeline). The MHPA sequencing data were deposited in the NCBI’s Database of Genotypes and Phenotypes (dbGaP; accession number phs002914.v1).

### Calculation of cosine similarity for mutation signatures.

The cosine similarity was calculated in R 4.1.0 using the cosine function from the lsa package.

### Calculation of the MHPA depth of coverage.

Calculation of the MHPA median depth of coverage per amplicon/sample was performed using Samtools depth command. Median coverage per amplicon was defined as the median depth of coverage for each genomic position in the queried sequence (targeted sequence, without primer-specific sequence). The depth of coverage (and VAF assessment) for genomic positions within the queried sequence that were overlapping with the primer-specific sequence of an adjacent amplicon, if present, was adjusted for this occurrence.

### Mutation review and annotation.

All variants identified in MHPA analysis were reviewed. For identification of SNVs, we applied a VAF threshold of ≥0.05%; for identification of indels/DNVs, we applied a VAF threshold of ≥0.01%. Each SNV/indel/DNV had to be supported by at least 4 consensus reads, representing 2 distinct UMI families. In addition, we used a Panel of Normals (PoNs) approach to filter out recurrent PCR errors and artifacts, using 2 PoNs: (a) *TSC2*-PoN (14 blood samples analyzed by *TSC2*-MHPA); and (b) *TP53*-PoN (5 non-sun-exposed skin samples — 2 TSC-NS, 2 TSC-nipple AF, and 1 TSC-UF — analyzed by *TP53*-MHPA). SNVs were filtered out if seen at VAF ≥ 0.025% in the aggregate PoNs; indels/DNVs were filtered out if seen at VAF ≥ 0.005% in the aggregate PoNs. Variants were retained, however, if the VAF was 5 times higher than seen in the PoNs.

All variants were manually reviewed in IGV (60). Artifacts (often in recurrent genomic positions), variants in misaligned reads, and indels in repetitive sequence tracts were discarded. Identification of adjacent variants in *cis*/in *trans* was also performed by manual review in IGV, looking at the entire extent of the affected amplicon. For overlapping SNVs/DNVs, the same VAF thresholds as for nonoverlapping SNVs/DNVs had to be met.

Human Genome Variation Society (HGVS) nomenclature was used for all variants (annotated using Mutalyzer 2.0.32; https://mutalyzer.nl) (62).

Several resources were used to assess the functional significance of somatic *TSC2* variants, including gnomAD (the population VAF reported in the total population for all *TSC2* variants; ref. 63), VEP (61), LOVD (25), ClinVar (64), and VarSome (65). Using these resources, the consensus functional significance of each *TSC2* variant was assigned: (a) “pathogenic” or “likely pathogenic”: pathogenic or likely pathogenic status in any of the resources and population VAF <0.01% in gnomAD; (b) variant of unknown significance (“VUS”): VUS status in any of the resources, without clear indication of pathogenicity in any other, and gnomAD AF <0.01%; (c) “likely benign”: likely benign or benign in any of the resources. Somatic *TSC2* mutations with gnomAD AF >0.01% (*n =* 8) were not considered as pathogenic, according to the corresponding status in other resources; for these variants either “VUS” or “likely benign” consensus status was assigned.

Seshat (66) was used for the assessment of functional significance of the identified somatic *TP53* variants.

### Calculation of the number of micro-FAFs in TSC facial skin.

The number of FAF clones in the facial skin of TSC patients was calculated as follows: (median number of somatic *TSC2* mutations per mosaic TSC-FAF) × [100/(median systemic mosaic VAF in FAFs × 2)] × (facial surface area)/(size of biopsy). The facial surface area corresponds to the facial region that is most often affected by FAF in TSC, i.e., (width: mean bizygomatic width) × [length: (mean upper facial height × 0.8) + (mean lower facial height × 0.33)], according to craniofacial measurements in ref. 67.

### Statistics.

Statistical analyses were performed using Prism 7 (v7.0d; GraphPad Software) and R (4.1.0). Specific statistical tests are indicated in the text. Fisher’s exact test and Kruskal-Wallis test (nonparametric unpaired test) with post hoc Dunn’s test for multiple-comparison correction were applied for comparison of the number of mutation subtypes in the analyzed subgroups of samples. Mann-Whitney test (nonparametric unpaired test) was used for comparison of mutation VAFs in hybrid-capture/amplicon MPS versus MHPA. For correlation analyses, Pearson’s coefficient and linear regression curve were generated. For mutation strand bias assessment, the binomial test was applied. *P* values less than 0.05 were considered statistically significant.

### Study approval.

All subjects included in our study provided written informed consent. All TSC subjects, except P13, were enrolled under the research protocols approved by our Institutional Review Board, the Human Research Committee of Mass General Brigham (2013P002667 and 2021P000046). Nipple AF biopsies from P13 were collected under the protocols approved by the NIH intramural Institutional Review Board (96-H-0100, 00-H-0051). The nonTSC-foreskin samples were collected under the research protocol approved by the Partners Human Research Committee (2021P003411).

## Author contributions

KK conceptualized the study, collected and performed DNA extraction from TSC and non-TSC samples, developed the MHPA strategy, designed and optimized the MHPA assays, generated the MHPA sequencing libraries with QC and quantification, participated in development of the custom computational pipeline for MHPA data analysis, performed computational analyses and interpreted the MPS data, collected and summarized clinical and demographic data, performed statistical analyses, wrote the manuscript, prepared all figures, tables, and supplementary materials, and acquired funding for the study. JMG collected and characterized most of the skin biopsy samples and performed clinical examination of the TSC and non-TSC patients. KG performed DNA extraction from TSC samples and generated and provided hybrid-capture and amplicon MPS data from TSC patients. BAO conceptualized the study and contributed to the MHPA strategy development. ZTH preprocessed the MHPA data and performed computational analyses. ART contributed to generation and analysis of MPS data. TND provided nipple AF samples and revised the manuscript. JM supported clinical evaluation and patients for nipple AF samples. DJK conceptualized and supervised the study, contributed to the MHPA strategy development, reviewed the MPS data, collected and reviewed clinical and demographic data, reviewed and participated in manuscript preparation together with KK, and acquired funding for the study. All authors read and commented on the paper.

## Supplementary Material

Supplemental data

ICMJE disclosure forms

Supplemental table 1

Supplemental table 2

Supplemental table 3

Supplemental table 4

Supplemental table 5

Supplemental table 6

## Figures and Tables

**Table 1:**
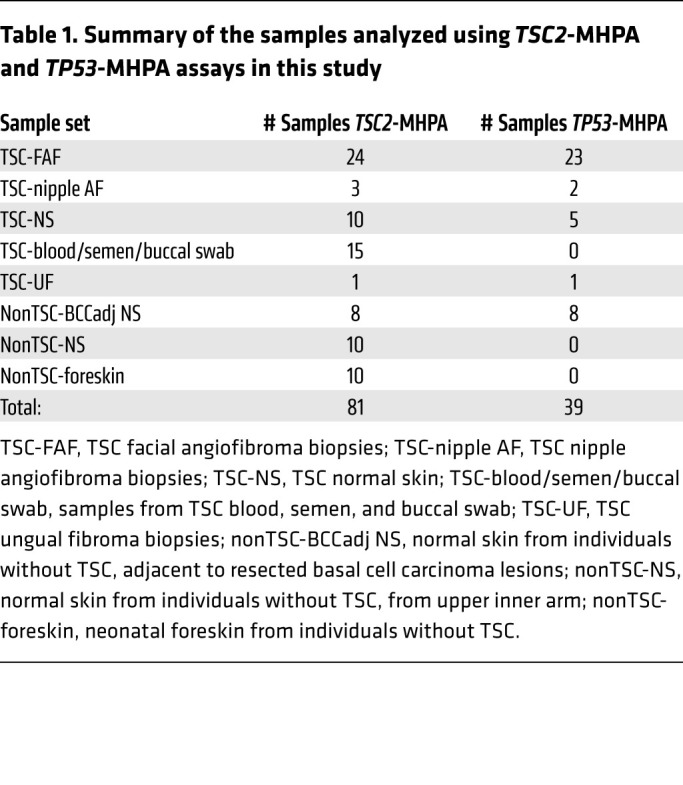
Summary of the samples analyzed using *TSC2*-MHPA and *TP53*-MHPA assays in this study

## Version 1 03/31/2022

In-Press Preview

## Version 2 05/16/2022

Electronic publication
