# Supplementary material for: Ultrasensitive profiling of UV-induced mutations identifies thousands of subclinical facial tumors in tuberous sclerosis complex
Source: J Clin Invest. 2022 May 16;132(10):e155858. doi: 10.1172/JCI155858 (PMC9106361; doi:10.1172/JCI155858)
Supplement: Supplemental data [file jci-132-155858-s182.pdf]

Supplemental Materials for

# Ultrasensitive profiling of UV-induced mutations identifies thousands of subclinical facial tumors in tuberous sclerosis complex

Katarzyna Klonowska*, Joannes M. Grevelink, Krinio Giannikou, Barbara A. Ogorek, Zachary T. Herbert, Aaron R. Thorner, Thomas N. Darling, Joel Moss, and David J. Kwiatkowski*

*Corresponding authors. e-mail: kklonowska@bwh.harvard.edu, dk@rics.bwh.harvard.edu

**This PDF file includes:**

Supplemental Figures 1 to 11

**Other Supplemental Materials for this manuscript include the following:**

Supplemental Tables 1 to 6

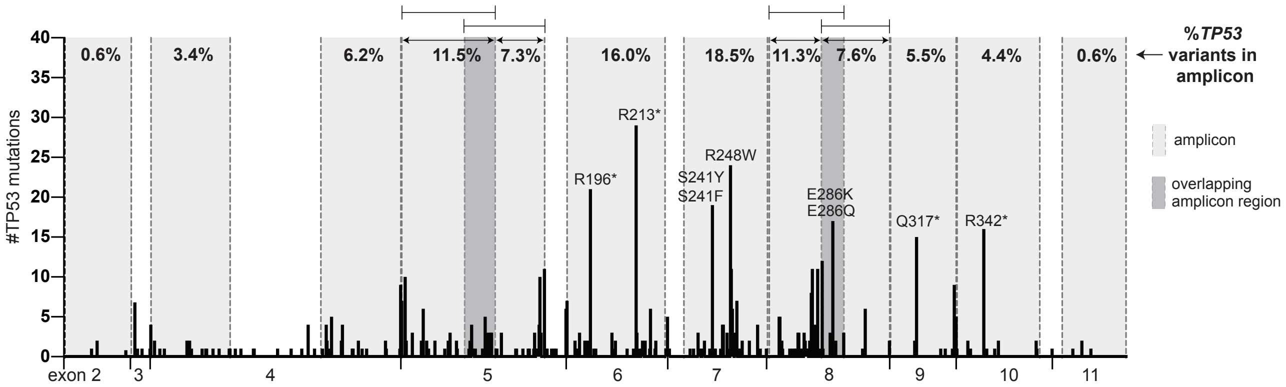


**Supplemental Figure 1.**
**Location of the *TP53*-MHPA amplicons on the map of nonsynonymous somatic *TP53* variants reported in normal skin and skin cancer.** Y-axis indicates number of *TP53* variants at the same nucleotide position. Variants are shown as vertical lines. Hotspots with variants reported > 15 times are labeled with amino acid position. Splice mutations are summed and shown as a single bar at each exon-exon junction. Genomic regions covered by MHPA assay amplicons are marked in light grey, with indication of the fraction of the variants covered by each of the amplicons (%). Amplicons in exon 5 and exon 8 are partially overlapping; the overlapped region is indicated in dark grey, as indicated in the legend.

A

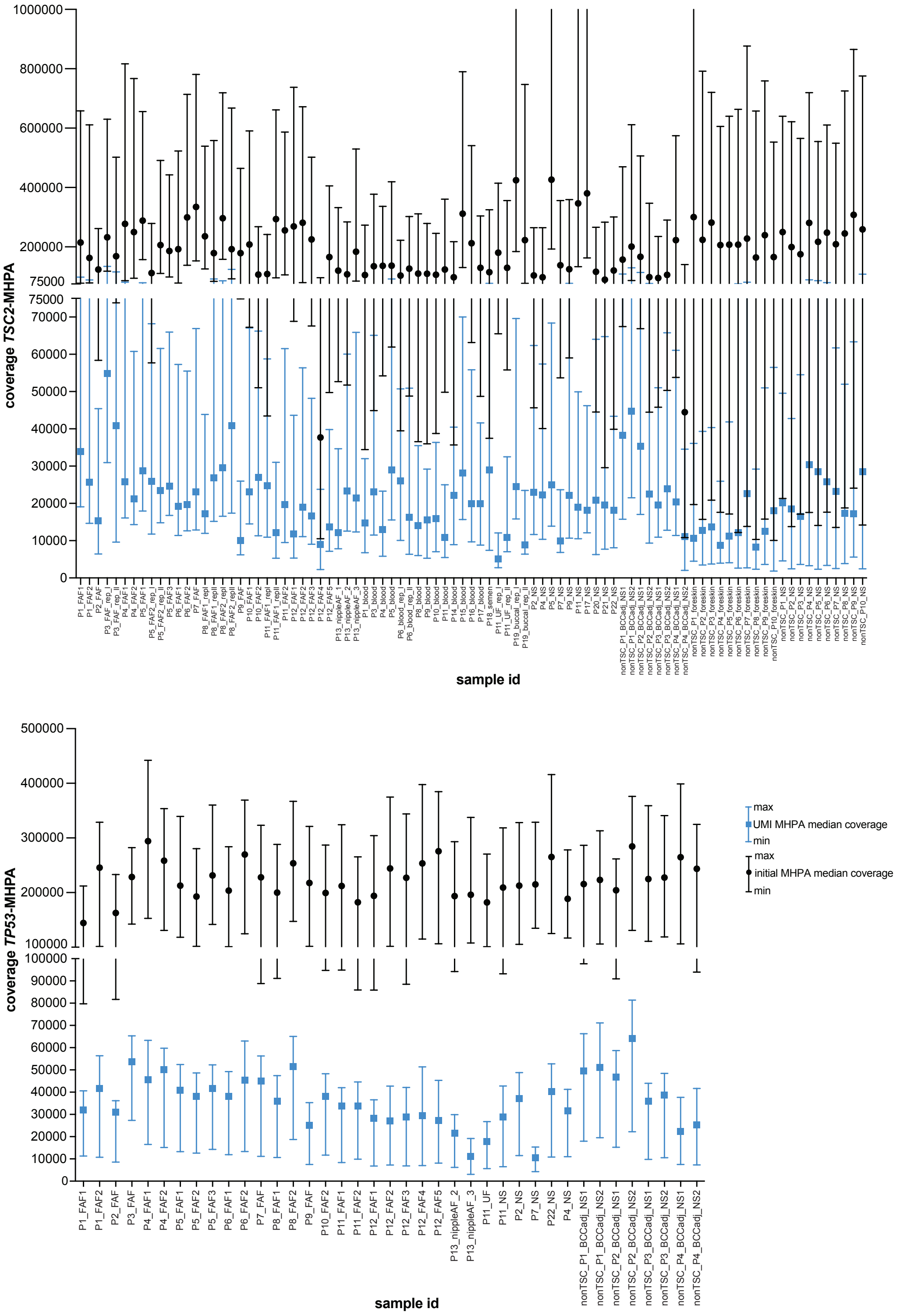
coverage TSC2-MHPA
1000000
800000
600000
400000
200000
75000
75000
70000
60000
50000
40000
30000
20000
10000
0
sample id
coverage TP53-MHPA
500000
400000
300000
200000
100000
100000
90000
80000
70000
60000
50000
40000
30000
20000
10000
0
P1_FAF1
P1_FAF2
P2_FAF
P3_FAF
P4_FAF1
P4_FAF2
P5_FAF1
P5_FAF2
P5_FAF3
P6_FAF1
P6_FAF2
P7_FAF
P8_FAF1
P8_FAF2
P9_FAF
P10_FAF2
P11_FAF1
P11_FAF2
P12_FAF1
P12_FAF2
P12_FAF3
P12_FAF4
P12_FAF5
P13_nippleAF_2
P13_nippleAF_3
P11_UF
P11_NS
P2_NS
P7_NS
P22_NS
P4_NS
nonTSC_P1_BCCadj_NS1
nonTSC_P1_BCCadj_NS2
nonTSC_P2_BCCadj_NS1
nonTSC_P2_BCCadj_NS2
nonTSC_P3_BCCadj_NS1
nonTSC_P3_BCCadj_NS2
nonTSC_P4_BCCadj_NS1
nonTSC_P4_BCCadj_NS2
sample id
max
UMI MHPA median coverage
min
max
initial MHPA median coverage
min

B

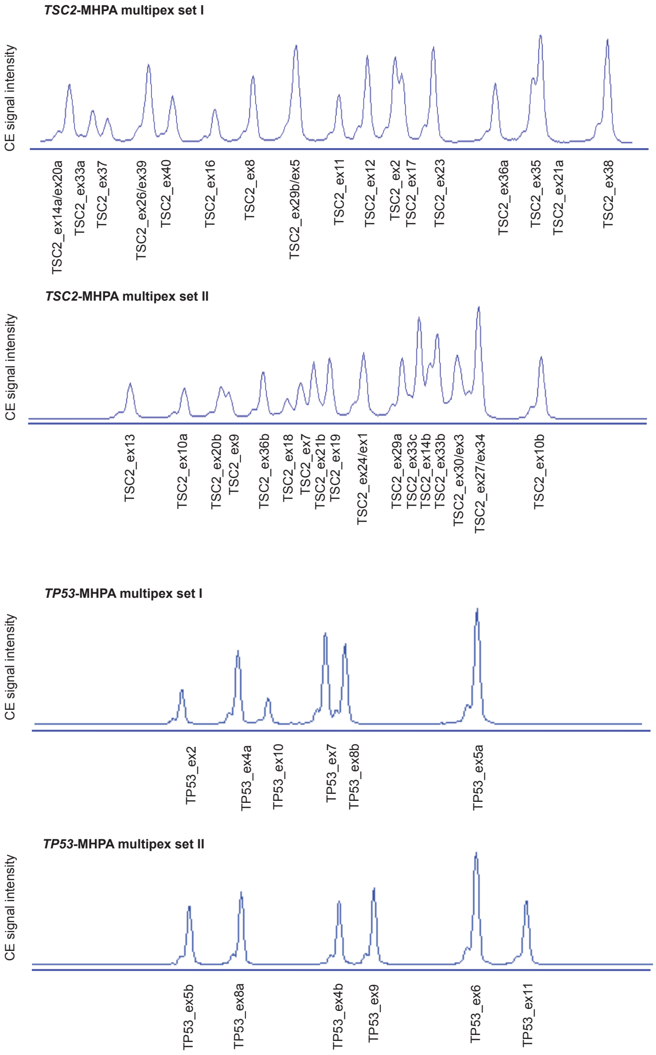

TSC2-MHPA multipex set I
CE signal intensity
TSC2_ex14a/ex20a
TSC2_ex33a
TSC2_ex37
TSC2_ex26/ex39
TSC2_ex40
TSC2_ex16
TSC2_ex8
TSC2_ex29b/ex5
TSC2_ex11
TSC2_ex12
TSC2_ex2
TSC2_ex17
TSC2_ex23
TSC2_ex36a
TSC2_ex35
TSC2_ex21a
TSC2_ex38
TSC2-MHPA multipex set II
CE signal intensity
TSC2_ex13
TSC2_ex10a
TSC2_ex20b
TSC2_ex9
TSC2_ex36b
TSC2_ex18
TSC2_ex7
TSC2_ex21b
TSC2_ex19
TSC2_ex24/ex1
TSC2_ex29a
TSC2_ex33c
TSC2_ex14b
TSC2_ex33b
TSC2_ex30/ex3
TSC2_ex27/ex34
TSC2_ex10b
TP53-MHPA multipex set I
CE signal intensity
TP53_ex2
TP53_ex4a
TP53_ex10
TP53_ex7
TP53_ex8b
TP53_ex5a
TP53-MHPA multipex set II
CE signal intensity
TP53_ex5b
TP53_ex8a
TP53_ex4b
TP53_ex9
TP53_ex6
TP53_ex11

C

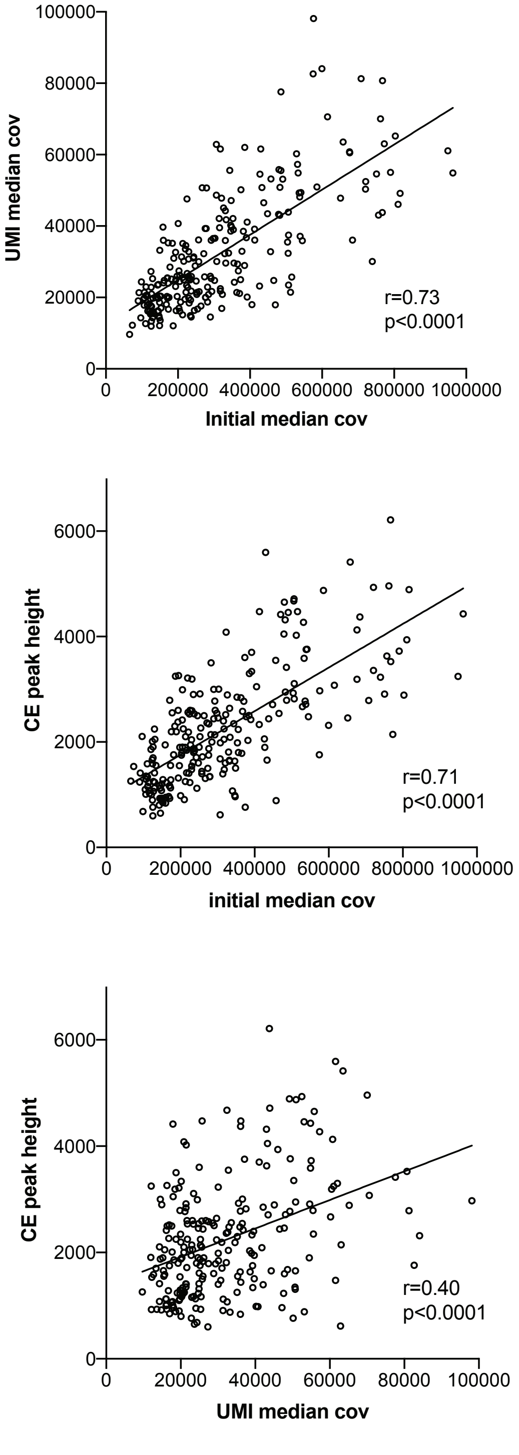
UMI median cov
Initial median cov
r=0.73
p<0.0001
CE peak height
initial median cov
r=0.71
p<0.0001
CE peak height
UMI median cov
r=0.40
p<0.0001

D

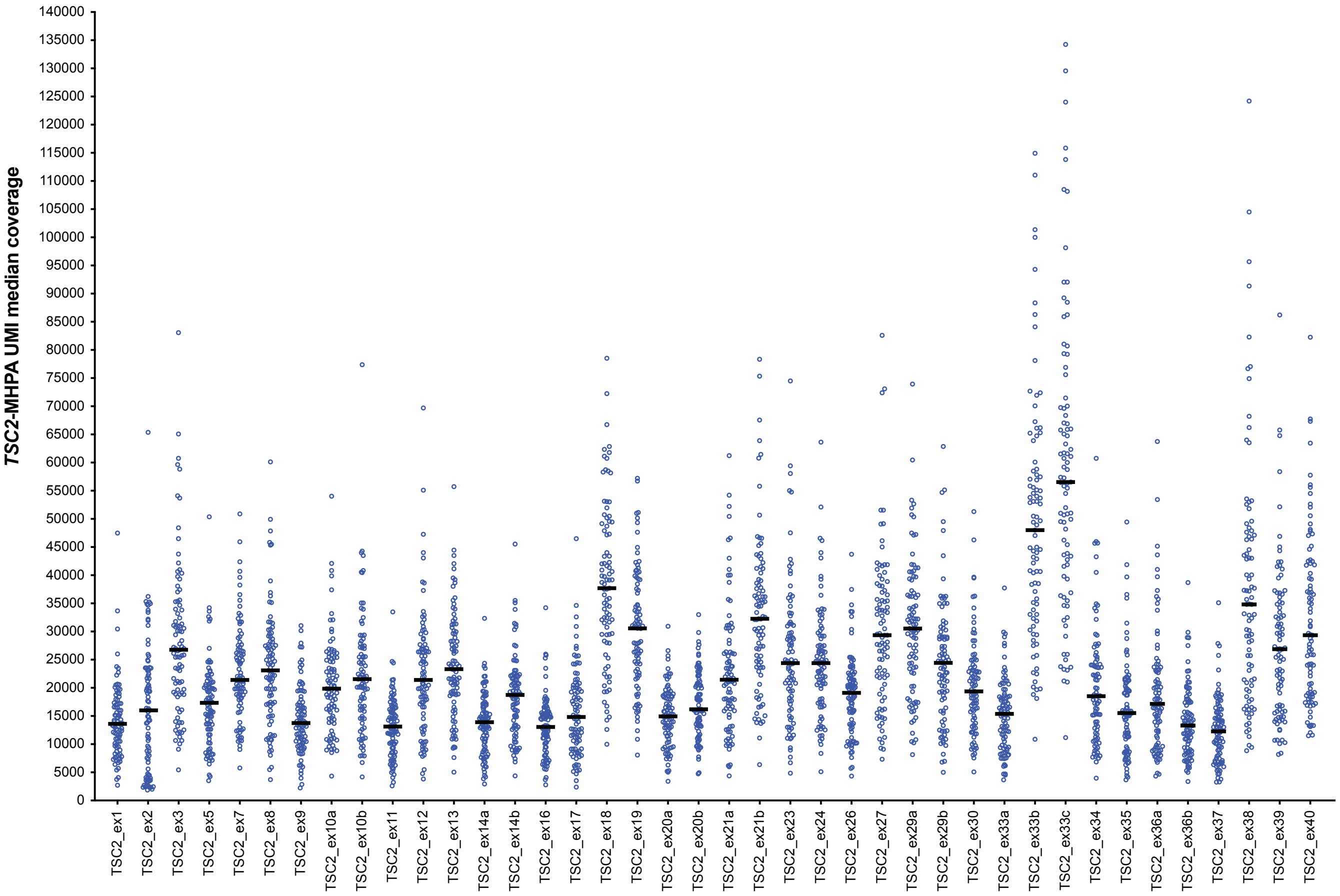

TSC2-MHPA UMI median coverage
140000
135000
130000
125000
120000
115000
110000
105000
100000
95000
90000
85000
80000
75000
70000
65000
60000
55000
50000
45000
40000
35000
30000
25000
20000
15000
10000
5000
0
TSC2_ex1
TSC2_ex2
TSC2_ex3
TSC2_ex5
TSC2_ex7
TSC2_ex8
TSC2_ex9
TSC2_ex10a
TSC2_ex10b
TSC2_ex11
TSC2_ex12
TSC2_ex13
TSC2_ex14a
TSC2_ex14b
TSC2_ex16
TSC2_ex17
TSC2_ex18
TSC2_ex19
TSC2_ex20a
TSC2_ex20b
TSC2_ex21a
TSC2_ex21b
TSC2_ex23
TSC2_ex24
TSC2_ex26
TSC2_ex27
TSC2_ex29a
TSC2_ex29b
TSC2_ex30
TSC2_ex33a
TSC2_ex33b
TSC2_ex33c
TSC2_ex34
TSC2_ex35
TSC2_ex36a
TSC2_ex36b
TSC2_ex37
TSC2_ex38
TSC2_ex39
TSC2_ex40


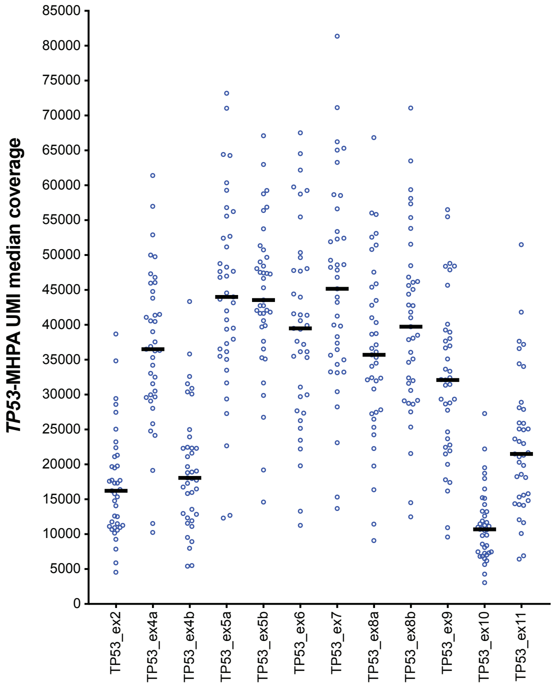

TP53-MHPA UMI median coverage
85000
80000
75000
70000
65000
60000
55000
50000
45000
40000
35000
30000
25000
20000
15000
10000
5000
0
TP53_ex2
TP53_ex4a
TP53_ex4b
TP53_ex5a
TP53_ex5b
TP53_ex6
TP53_ex7
TP53_ex8a
TP53_ex8b
TP53_ex9
TP53_ex10
TP53_ex11

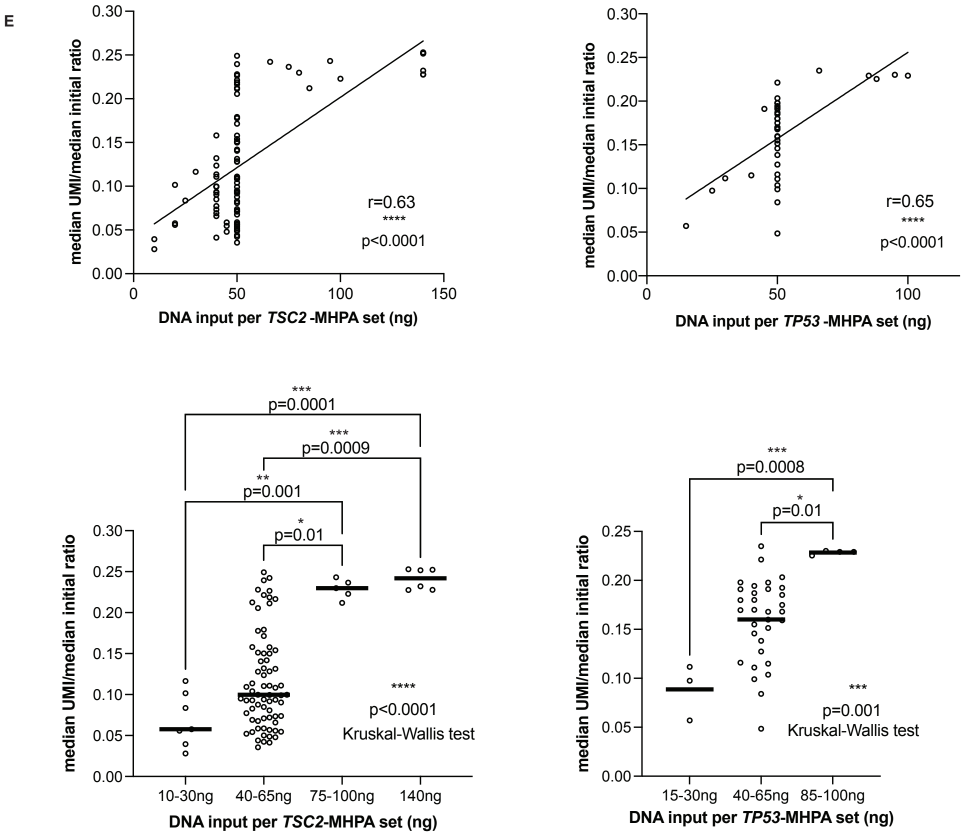


**Supplemental Figure 2.**

**Depth of coverage for *TSC2*-MHPA and *TP53*-MHPA assays and capillary electrophoresis QC. (A)** Summary of the median depth of coverage for samples subject to *TSC2*-MHPA and *TP53*-MHPA analyses. Min (corresponding to the amplicon with the smallest median depth in a given sample), median (corresponding to the median depth across amplicons) and max (corresponding to the amplicon with the highest median depth in a given sample) are presented for each sample, before ('initial') and after ('UMI') UMI compression **(B)** Capillary electrophoresis (CE) separation of amplicons from *TSC2*-MHPA and *TP53*-MHPA assays (separately for SETI and SETII, for the respective assays). For *TSC2*-MHPA, CE signals of some of the amplicons (i.e., SETI: TSC2_ex26/ex39, TSC2_ex28b/ex5; SETII: TSC2_ex24/ex1, TSC2_ex30/ex3; TSC2_ex27/ex34 ) overlap and are represented by one common peak, because of overlapping peaks. The CE spectra for both *TSC2*-MHPA and *TP53*-MHPA demonstrate uniform multiplex amplification of the amplicons in the respective SETs/assays. **(C)** Correlation between CE signal intensity and MPS median depth for each *TSC2*-MHPA amplicon, for a set of 7 representative samples. Top: correlation between corresponding median MPS depth of coverage before ('initial median cov') and after ('UMI median cov') UMI consensus compression. Middle: correlation between corresponding median MPS depth of coverage before UMI consensus compression and CE signal intensity. Bottom: correlation between corresponding median MPS depth of coverage after UMI consensus compression and CE signal intensity. For the pairs of the overlapping amplicons represented by common peaks [see **(B)**], median MPS depth values have been summed for the purpose of correlation analysis. R represents Pearson correlation coefficient and is presented along with two-sided p-value. The curve was generated using linear regression. **(D)** Summary of median coverage for each of the *TSC2*-MHPA and *TP53*-MHPA amplicons, across all analyzed samples. The horizontal black bar represents median coverage across all

samples, for a given amplicon. **(E)** Correlation analysis of DNA input and the sequencing coverage after UMI consensus compression. Y-axis represents the ratio of median depth of coverage in the .bam files before and after UMI compression for each sample. A lower ratio reflects a greater degree of compression by the UMI process. R represents Pearson correlation coefficient and is presented along with two-sided p-value. The curve was generated using linear regression. The bottom graph is the same data as above, but each sample is placed into one of four groups. P-values for the pairwise comparisons within multiple groups were adjusted for multiple comparisons using post-hoc Dunn’s test, performed along with Kruskal-Wallis test. The horizontal bars indicate median values.

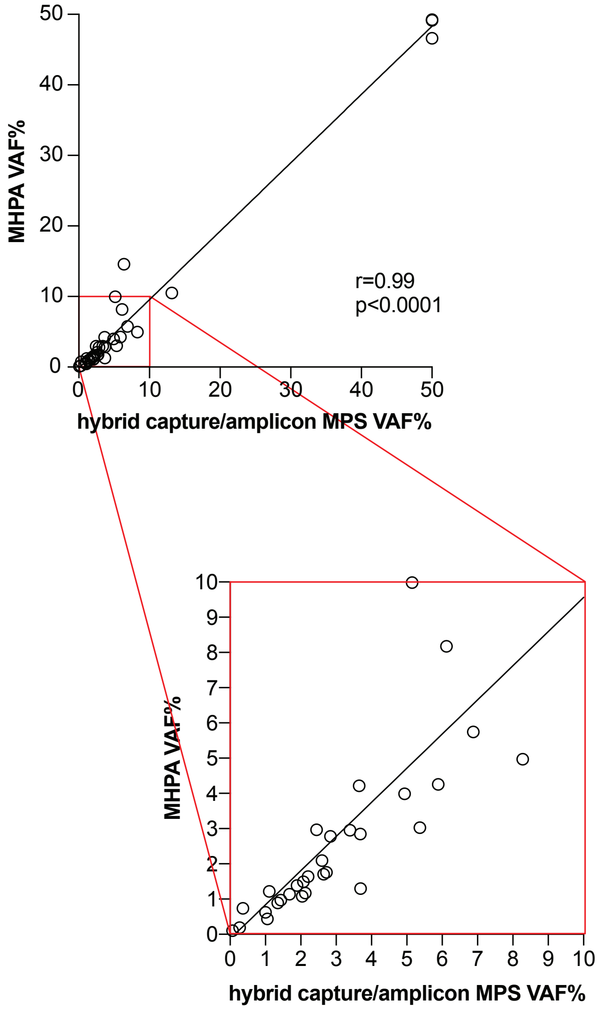


**Supplemental Figure 3.**
**Correlation of VAFs from prior hybrid capture or amplicon MPS and *TSC2*-MHPA analysis.**

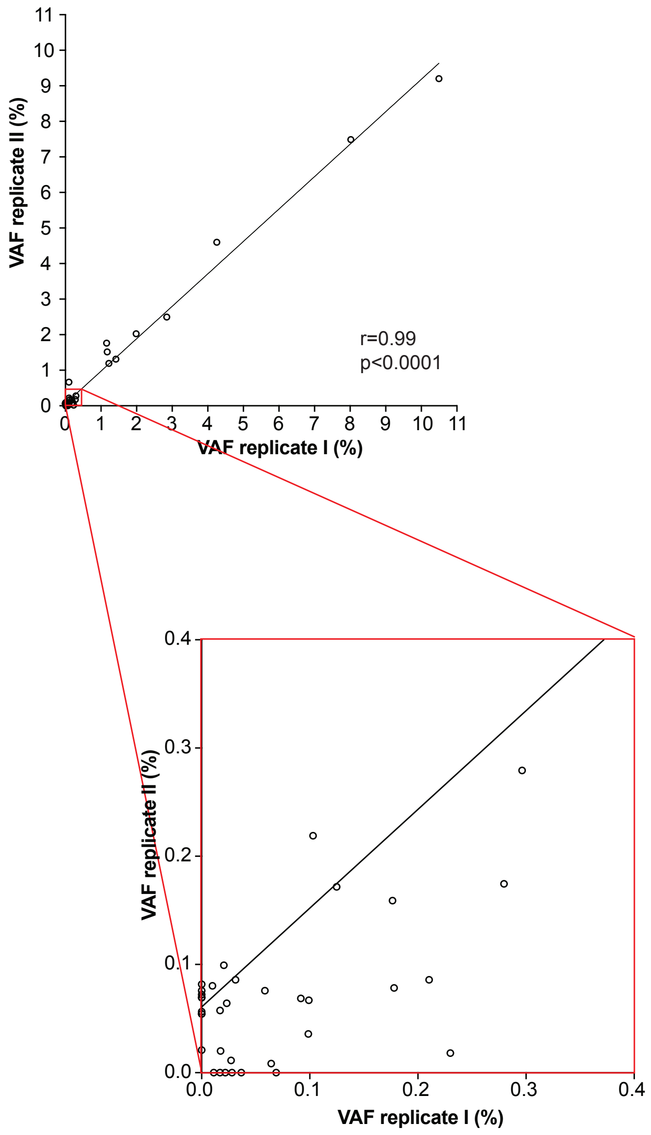


**Supplemental Figure 4.**

**Correlation analysis of VAFs in MHPA replicates (i.e., 8 samples for which MHPA analysis was performed twice).**

A

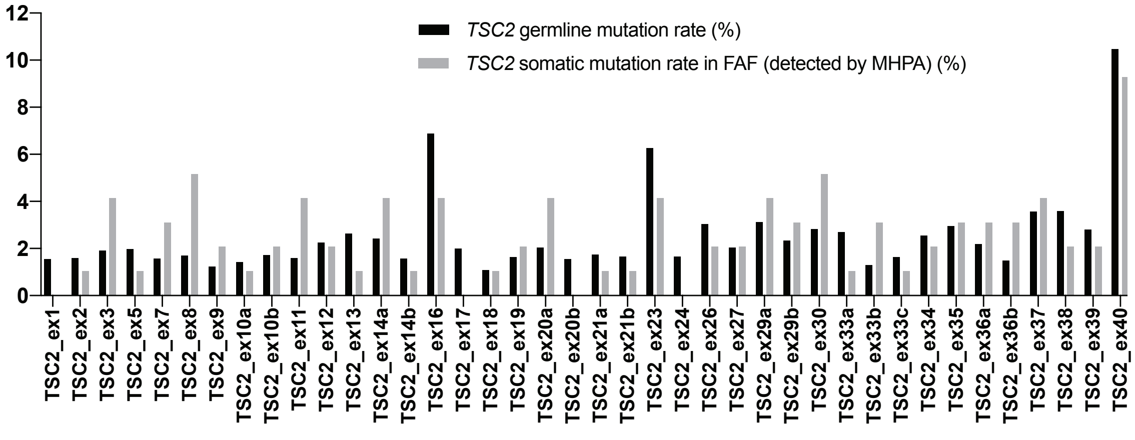

TSC2 germline mutation rate (%)
TSC2 somatic mutation rate in FAF (detected by MHPA) (%)
12
10
8
6
4
2
0
TSC2_ex1
TSC2_ex2
TSC2_ex3
TSC2_ex5
TSC2_ex7
TSC2_ex8
TSC2_ex9
TSC2_ex10a
TSC2_ex10b
TSC2_ex11
TSC2_ex12
TSC2_ex13
TSC2_ex14a
TSC2_ex14b
TSC2_ex16
TSC2_ex17
TSC2_ex18
TSC2_ex19
TSC2_ex20a
TSC2_ex20b
TSC2_ex21a
TSC2_ex21b
TSC2_ex23
TSC2_ex24
TSC2_ex26
TSC2_ex27
TSC2_ex29a
TSC2_ex29b
TSC2_ex30
TSC2_ex33a
TSC2_ex33b
TSC2_ex33c
TSC2_ex34
TSC2_ex35
TSC2_ex36a
TSC2_ex36b
TSC2_ex37
TSC2_ex38
TSC2_ex39
TSC2_ex40


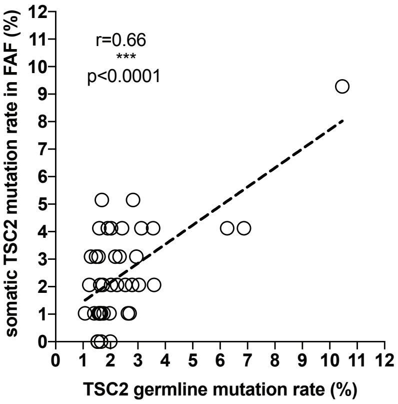

r=0.66
***
p<0.0001
somatic TSC2 mutation rate in FAF (%)
TSC2 germline mutation rate (%)
0 1 2 3 4 5 6 7 8 9 10 11 12

B

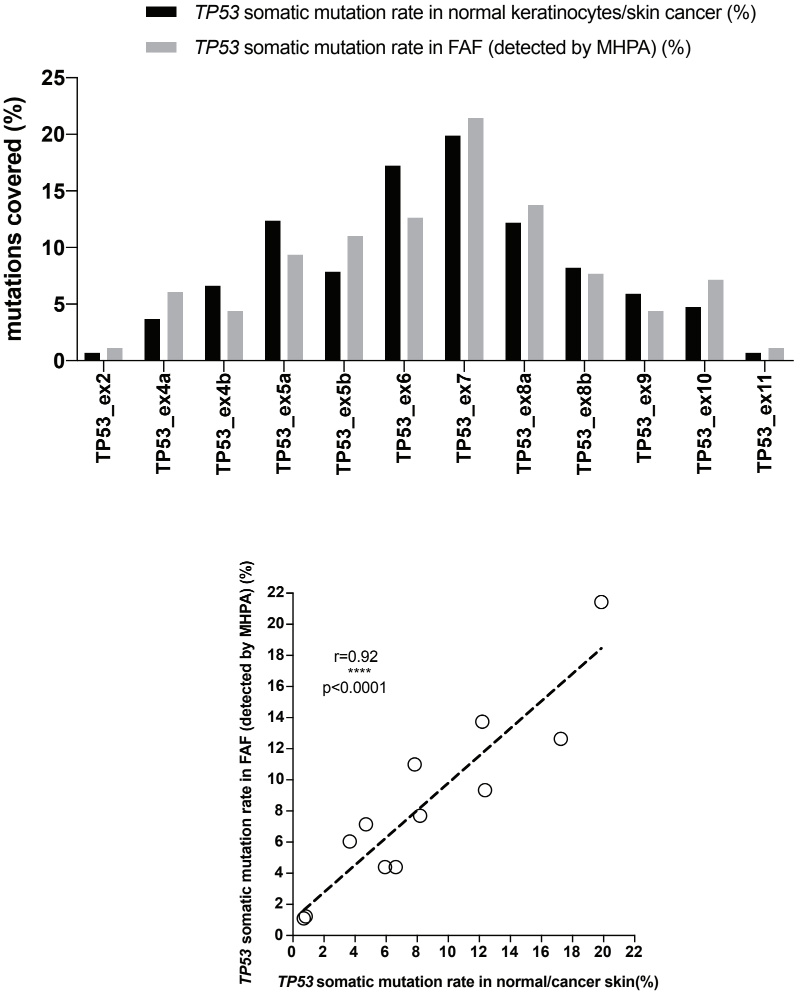


**Supplemental Figure 5.**

**Comparison of *TSC2* (A) and *TP53* (B) mutation prevalence by exon from prior germline studies (*TSC2*) and prior mutation analyses (*TP53*), in comparison to MHPA analysis of FAFs.** Top, graph of all exons. Bottom, correlation plot. Specific fractions (%) are indicated in Supplemental Table 1. r – Pearson correlation coefficient; p – two-sided p-value.

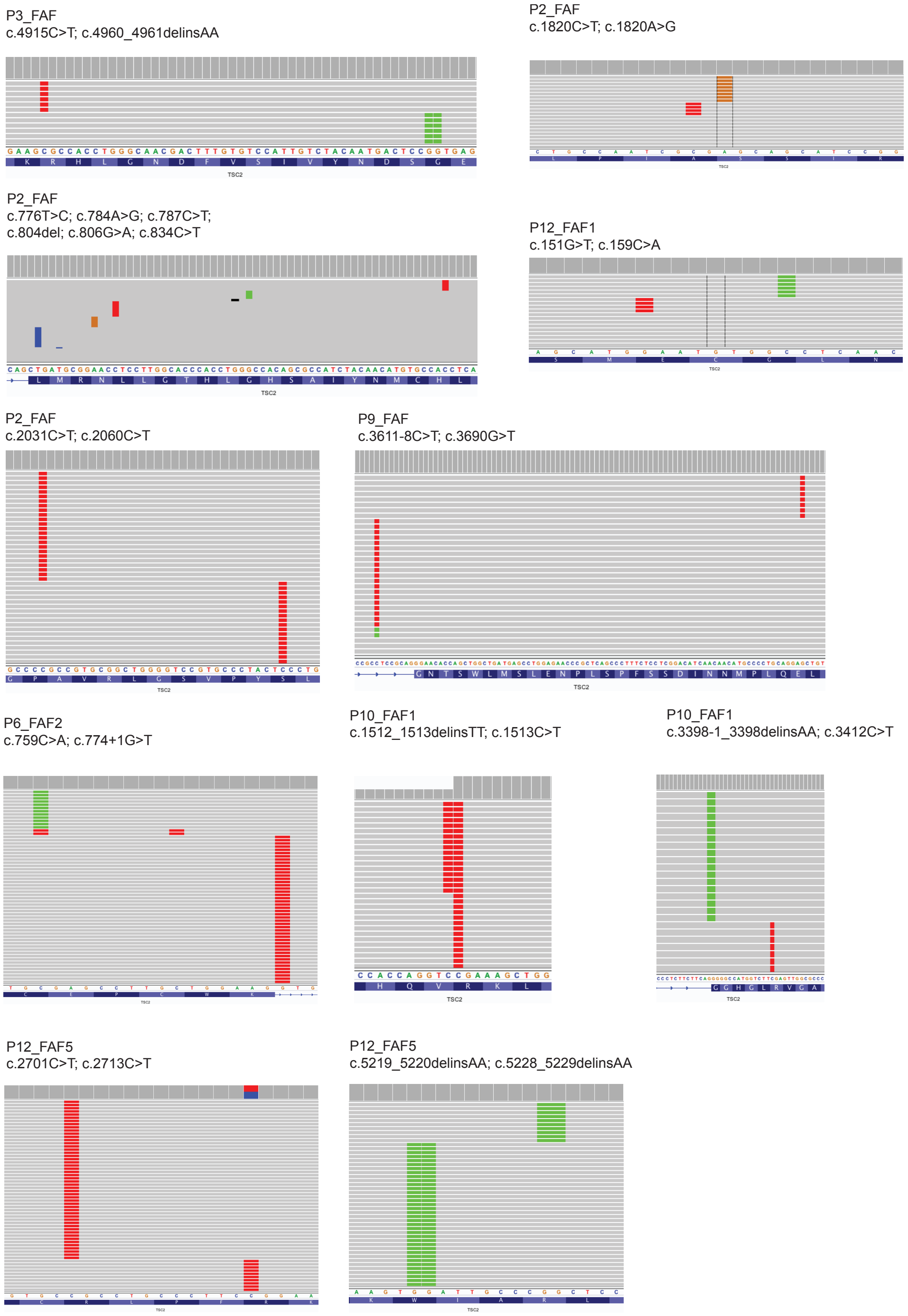


**Supplemental Figure 6.**

**IGV snapshots of *TSC2* mutations occurring in close vicinity *in trans*, within single MHPA *TSC2* amplicons.**

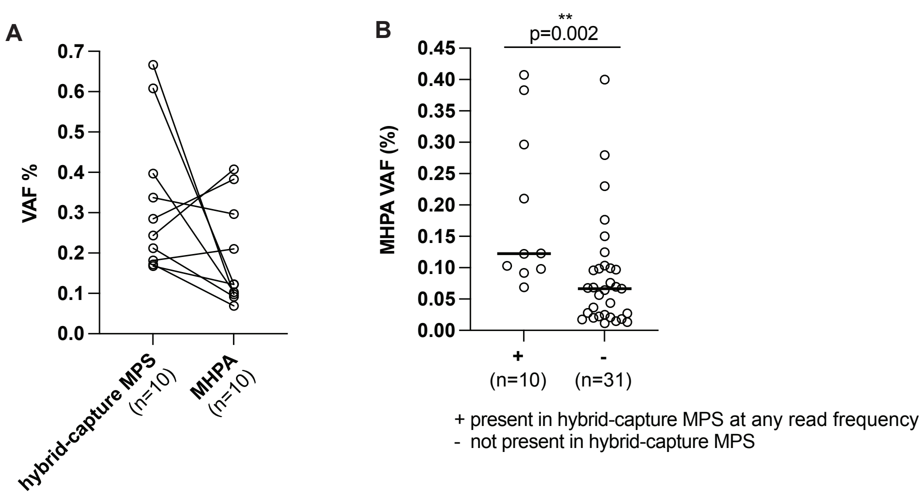


**Supplemental Figure 7.**

***TSC2* somatic variants newly identified by MHPA, compared with prior hybrid capture MPS data. (A)** Variants seen in both MHPA and prior MPS. The interconnected dots indicate VAFs for the same samples. **(B)** MHPA VAFs for mutations seen (+) and not seen (-) in prior hybrid capture MPS (Mann-Whitney test, two-sided p-value). The horizontal bars indicate median values.

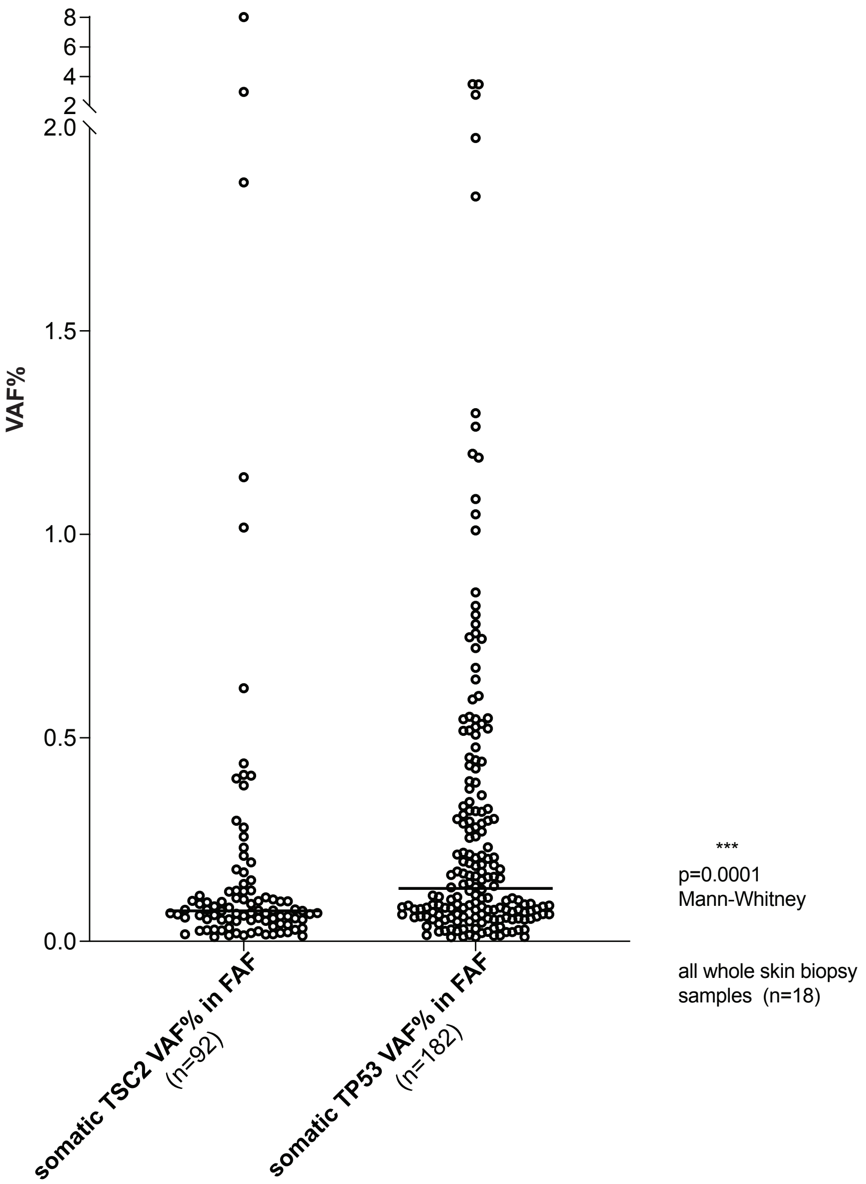


**Supplemental Figure 8.**

**Comparison of the VAFs of somatic *TSC2* and *TP53* mutations in TSC-FAF.** The horizontal bars indicate median values.

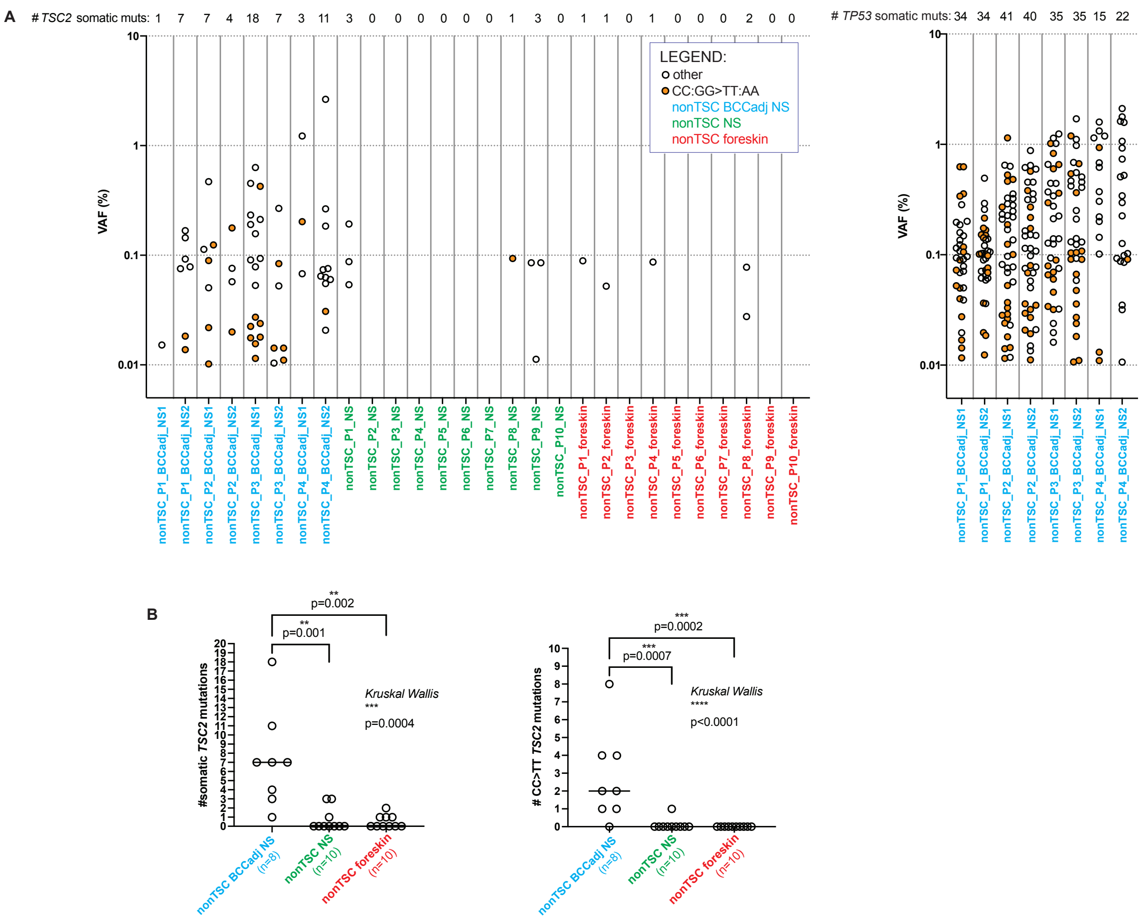

**Supplemental Figure 9.**
**Summary of *TSC2* and *TP53* mutations identified using MHPA in nonTSC skin samples. (A)** Circles filled with orange correspond to CC:GG>TT:AA mutations, while plain circles indicate other mutations. Y-axis indicates the variant allele frequencies (VAFs), while x-axis indicates sample labels. Abbreviations: '# somatic muts' – number of somatic mutations observed in each sample. **(B)** Comparison of the number of all somatic (left-hand side) and CC>TT only (right-hand side) mutations in nonTSC-BCCadj NS, nonTSC-NS and nonTSC-foreskin. P-values for the pairwise comparisons within multiple groups were adjusted for multiple comparisons using post-hoc Dunn's test, performed along with Kruskal-Wallis test. For pairwise comparisons, only significant (<0.05) p-values are indicated. The horizontal bars indicate median values.

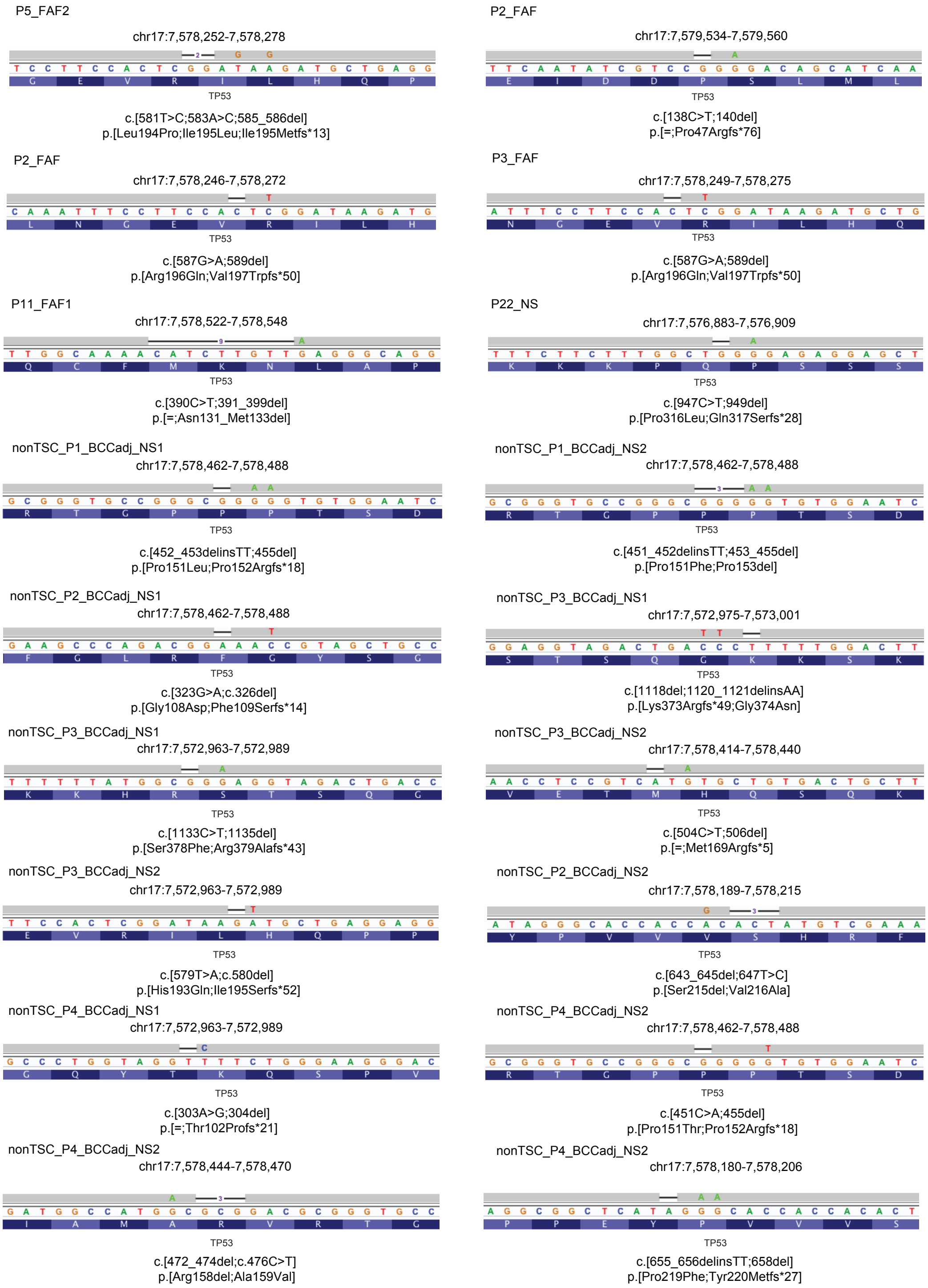


**Supplemental Figure 10.**
**IGV screenshots for each of the adjacent indel-SNVs/DNVs identified in *TP53* using MHPA.**

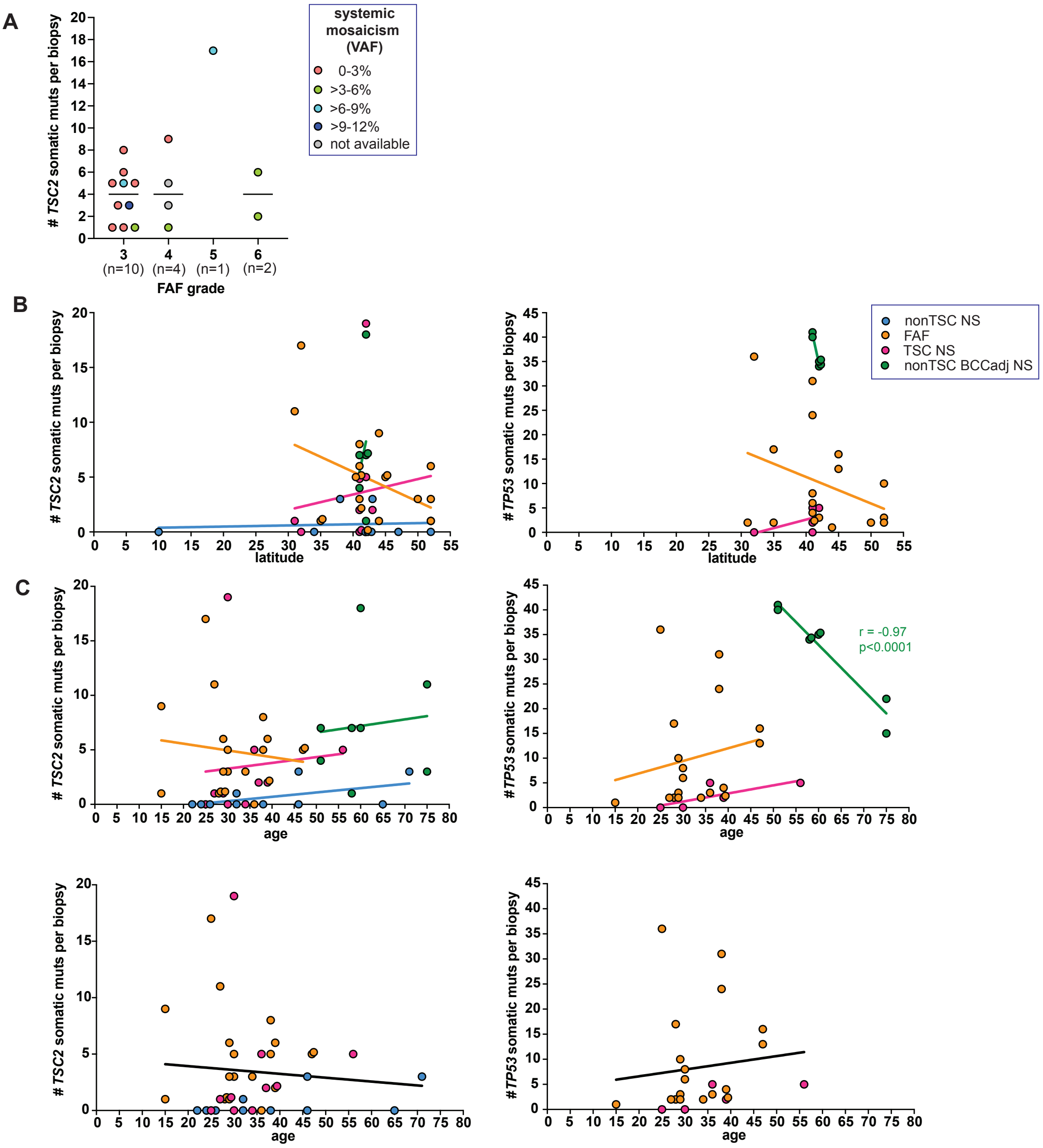
A
B
C
# TSC2 somatic muts per biopsy
FAF grade
3 (n=10)
4 (n=4)
5 (n=1)
6 (n=2)
systemic mosaicism (VAF)
0-3%
>3-6%
>6-9%
>9-12%
not available
# TP53 somatic muts per biopsy
latitude
nonTSC NS
FAF
TSC NS
nonTSC BCCadj NS
age
r = -0.97
p<0.0001

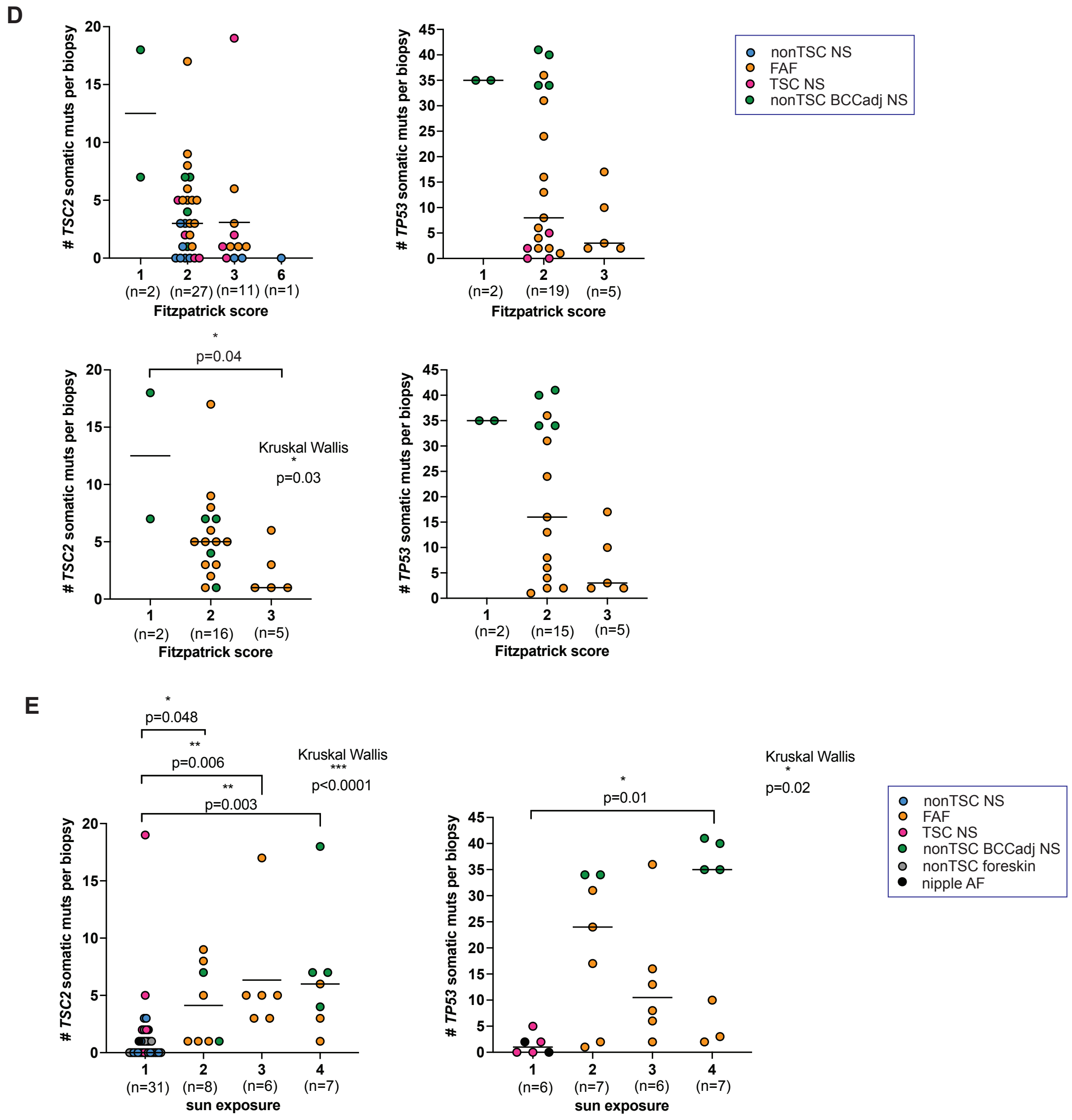


**Supplemental Figure 11. Somatic mutation prevalence and clinical characteristics of skin biopsies and their donors. (A)** Comparison of the number of *TSC2* somatic mutations in FAF biopsies with different FAF grades (FASI grade, see Supplemental Table 4). The range of VAF of the systemic mosaic *TSC2* mutations is color coded for each of the biopsies, as indicated in the legend. **(B)** Number of *TSC2* (left) and *TP53* (right) somatic mutations in skin biopsies from donors living at different latitudes. **(C)** Correlation analysis of the number of somatic *TSC2* (left) and *TP53* (right) mutations and skin biopsy donors' age. Top row: Linear regression curves for different skin biopsy subgroups are color-coded as indicated in the legend. Bottom row: Black linear regression curve was generated for all data point in the plots. Data for nonTSC-BCCadj NS samples were removed in the bottom row to avoid confounding due to their high mutation rate likely due to high UV exposure. **(D)** Number of somatic *TSC2* and *TP53* mutations in skin biopsies from donors with different Fitzpatrick skin type (see Supplemental Table 4). Top row: all skin biopsy samples studied except foreskin, UF and nipple FAF. Bottom row: only samples subject to high sun exposure (FAF, nonTSC-BCCadj NS). **(E)** Comparison of the number of *TSC2* and *TP53* somatic mutations in skin biopsies with different sun exposure (see Supplemental Table 4).
Skin biopsy subgroups are color coded as indicated. P-value is indicated for significant comparisons only (either correlation or Kruskal-Wallis test analysis). In **(B)-(C),** no

correction for multiple testing was performed. In **(A)** and **(D)-(E)**, p-values for the pairwise comparisons within multiple groups were adjusted for multiple comparisons using post-hoc Dunn's test, performed along with Kruskal-Wallis test. Overlapping data points on some plots **(B)-(C)** were shifted slightly for better visualization. The horizontal bars indicate median values.
